# Development and initial testing of a multi-stakeholder intervention for Lynch syndrome cascade screening: an intervention mapping approach

**DOI:** 10.1186/s12913-022-08732-6

**Published:** 2022-11-24

**Authors:** Lauren Passero, Swetha Srinivasan, Mary E. Grewe, Jennifer Leeman, Jonathan Berg, Daniel Reuland, Megan C. Roberts

**Affiliations:** 1 Division of Pharmaceutical Outcomes and Policy, UNC Eshelman School of Pharmacy, University of North Carolina at Chapel Hill, NC Chapel Hill, US; 2 North Carolina Translational and Clinical Sciences Institute, University of North Carolina at Chapel Hill, NC Chapel Hill, US; 3 School of Nursing, University of North Carolina at Chapel Hill, NC Chapel Hill, US; 4 School of Medicine, University of North Carolina at Chapel Hill, NC Chapel Hill, US

**Keywords:** Lynch syndrome, Intervention design, Intervention mapping, Cascade screening, Usability testing

## Abstract

**Background:**

Lynch syndrome is an underdiagnosed hereditary condition carrying an increased lifetime risk for colorectal and endometrial cancer and affecting nearly 1 million people in the United States. Cascade screening, systematic screening through family members of affected patients, could improve identification of Lynch syndrome, but this strategy is underused due to multi-level barriers including low knowledge about Lynch syndrome, low access to genetics services, and challenging family dynamics.

**Methods:**

We used intervention mapping, a 6-step methodology to create stakeholder-driven interventions that meet the needs of a target population, to develop an intervention to improve cascade screening for Lynch syndrome. The intervention development process was guided by input from key stakeholders in Lynch syndrome care and patients. We conducted usability testing on the intervention with Lynch syndrome patients using qualitative semi-structured interviewing and rapid qualitative analysis.

**Results:**

We developed a workbook intervention named *Let’s Talk* that addresses gaps in knowledge, skills, self-efficacy, outcome expectancy and other perceived barriers to cascade screening for Lynch syndrome. *Let’s Talk* contained educational content, goal setting activities, communication planning prompts and supplemental resources for patients to plan family communication. Evidence-based methods used in the workbook included information chunking, guided practice, goal setting and gain-framing. We conducted usability testing focused on the complexity and relative advantage of the intervention through 45-min virtual interviews with 10 adult patients with Lynch syndrome recruited from a national advocacy organization in the United States. Usability testing results suggested the intervention was acceptable in terms of complexity and relative advantage to other available resources, but additional information for communication with young or distant family members and a web-based platform could enhance the intervention’s usability.

**Conclusions:**

Intervention mapping provided a framework for intervention development that addressed the unique needs of Lynch syndrome patients in overcoming barriers to cascade screening. Future work is needed to transform *Let’s Talk* into a web-based tool and evaluate the effectiveness of the intervention in clinical practice with patients and genetic counselors. Intervention mapping can be useful to researchers as an evidence-based technique to develop stakeholder-centered interventions for addressing the needs of other unique populations.

**Supplementary Information:**

The online version contains supplementary material available at 10.1186/s12913-022-08732-6.

## Background

An estimated 1 million people in the United States live with Lynch syndrome (LS)—a genetic condition that carries an increased lifetime risk for several cancers including a lifetime risk of up to 82% for colorectal and 60% for endometrial cancers [1], compared to 4% [2] and 3% [3] in the general population, respectively. As such, LS research has been recognized as a national priority by HealthyPeople 2020 [4] and the Cancer Moonshot Blue Ribbon panel [5]. Universal tumor testing for biomarkers commonly found in LS cancers and follow-up germline genetic testing (when indicated) for patients with endometrial or colorectal cancer [6] can identify those with LS and reduce the morbidity and mortality of this syndrome through guideline recommended risk management strategies [7–10]. Opportunities for identifying individuals with LS before a cancer diagnosis include genetic testing among relatives of individuals diagnosed with LS through a process called “cascade screening” [11].

Cascade screening can reduce the cancer burden associated with LS. Given that it is an autosomal dominant condition, first degree relatives of those with LS have a 50% chance of also having the syndrome [1]. A recent public health impact report found that over half of colorectal cancer deaths associated with LS (~ 6,500 deaths per year) could be avoided if family members were identified through cascade screening and received subsequent risk management [6, 12]. As such, clinical guidelines recommend genetic counseling and testing for at-risk family members.

Despite guideline recommendations, cascade screening for LS is underused. It has been estimated that more than 98% of people with LS are unaware that they have LS [13]. While clinical guidelines recommend genetic counseling and testing for at-risk family members, little guidance is provided on how to implement this evidence-based practice. A recent literature review suggested that only 52% or fewer first-degree family members of individuals with LS receive cascade screening [14]. Multilevel barriers to cascade screening include: deficits in physicians’ knowledge and understanding about LS, challenging family dynamics, lack of follow-up during cascade screening; and access to providers of genetic services. Provider referral remains a strong predictor of uptake of genetic counseling and testing in several hereditary disorders [15–17], and patients have reported a desire for clinicians to be involved when sharing information with family members [18]. Thus, both patients and clinicians likely hold an important role to improve cascade screening.

Using input from key stakeholders, intervention mapping is a systematic, 6-step, stakeholder-engaged process for developing an intervention that addresses the needs of a targeted population by clearly describing health problems and developing acceptable, theory-based methods that support the adoption of the intervention and result in the desired health outcomes [19, 20]. This method has been used to develop effective interventions to improve health-related outcomes [21–24]; however, intervention mapping has not been used to develop interventions to improve the uptake of cascade screening. In fact, few interventions have been developed and tested to improve cascade screening for LS specifically [25]. By emphasizing the needs of key stakeholders, the intervention mapping methodology is well-positioned to address the multilevel barriers to cascade screening. The objective of this paper is to describe how we used intervention mapping to develop an intervention to improve cascade screening for LS.

## Methods

**Fig. 1:**
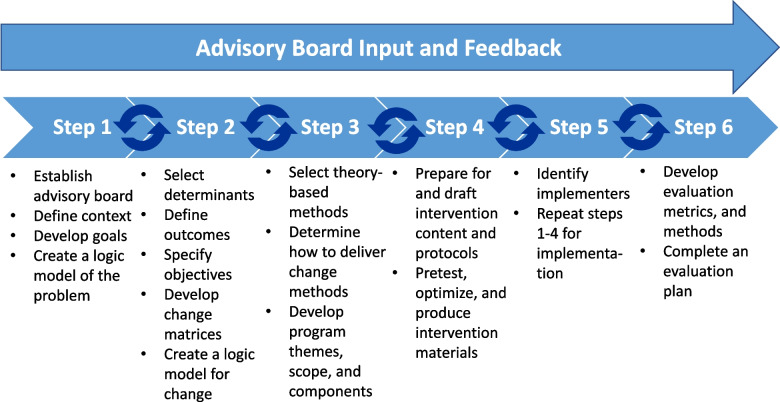
Intervention mapping process

First, we developed an advisory board consisting of a patient, genetics providers, an oncologist, a primary care provider, a patient advocate and methodological experts, to provide input throughout the six-step, intervention development process. Key tasks for each step are described below and in Fig. 1.

STEP 1: After establishing the advisory board, we conducted a needs assessment to understand the major barriers to and facilitators for cascade screening for LS. This consisted of synthesizing data from the authors’ previously published qualitative interviews with 60 patients, providers, and administrators [26] and systematic review [27]. These data were used in this phase of intervention mapping to [1] identify determinants of cascade screening, [2] develop a logic model of the problem, [3] determine the population and setting for the intervention, and [4] establish intervention goals. Two members of the team (MCR and SS) developed a logic model to synthesize data on key determinants.

STEP 2: From the advisory board, several key stakeholders (a patient, two genetic counselors, a primary care provider, and an implementation scientist) independently rated determinants according to their perceived importance for increasing cascade screening rates and changeability in the complex multilevel environment of cascade screening. Findings were summarized, shared with the full advisory board and used to inform intervention goals for changing the behavior of LS patients, family members, and providers (e.g., increased communication of LS information from the patient to family member). Two members of the research team (MCR and SS) then broke these behavioral outcomes down into specific performance objectives to outline what specific actions participants would need to accomplish to meet our goals. We mapped these specific performance objectives to the prioritized determinants to identify the precise changes (i.e., a change objective) needed to achieve our intervention goals. For example, if knowledge about LS was a key barrier among patients (i.e., determinant), and we want patients to contact relatives who are at risk for LS (i.e., performance objective), then patients must understand how to identify which family members are at risk for LS (a change objective). We developed these objectives for patients, family members and the genetic counselor (or provider who returns LS results). In parallel, a second group of behavioral researchers independently examined our systematic review data to develop a logic model of change using the PRECEDE-PROCEED model [28]. Their logic model was compared to ours to assess reliability of our model. Furthermore, a genetic counselor and an implementation scientist from our advisory board reviewed the performance and change objectives selected for each user type.

STEP 3: Informed by work in Steps 1 and 2, we developed our intervention’s scope and major themes. We then selected theory-based change methods (e.g., information chunking) to address our change objectives (e.g., increase knowledge about LS among patients), and we also chose practical applications (e.g., fact sheet) to deliver these methods. By the end of this step, we had a deep understanding of the components of our intervention, including the methods and materials needed which we shared with health behavioral scientists from our advisory board for feedback.

STEP 4: The intervention components developed in Step 3 were then designed. We developed materials (e.g., educational materials), messages and an intervention protocol. We also pretested, refined and produced our materials during this stage with support from the UNC Connected Health Applications and Interventions (CHAI) Core. Pretesting occurred in two stages. First, select members of the advisory board reviewed and provided feedback on the main intervention components, study protocol, and feasibility study design (see also Steps 5 and 6). Preliminary changes were made to the protocol and intervention.

Second, the refined intervention was presented to four genetic counselors (including two genetic counselors involved in rating determinants in Step 2) and a patient advocate on the advisory board. Specifically, we asked for feedback on the following areas:

[1] *In general, what do you like or dislike about this version of Let’s Talk* [the intervention]*? (a) How did you find the length of the workbook? (b) How did you find the organization of the workbook sections? (c) Was there any part of the workbook you found particularly difficult to understand or follow? (d) What aspects did you like or dislike about the paper format of the workbook? (e) Does the format of the workbook create any barriers to the successful completion of the workbook exercises?* and [2] *What parts of Let’s Talk would you change in a future version? What parts of Let’s Talk would you keep in a future version?*

Following this feedback, we made additional revisions to the look and content of the intervention.

### Qualitative Usability Testing Methods.

We then conducted a usability study with 10 individuals with LS. We identified adults (age 18 or older) through a Facebook advertisement on Lynch Syndrome International’s closed Facebook group [29] to reach participants across the United States. We delivered a copy of the intervention materials to participants via email after conducting verbal informed consent interviews and asked them to independently review the content and design. We (LP and MEG) conducted 45-min follow up interviews approximately two weeks after participants received the intervention to understand perceptions of the intervention and identify recommendations for improving the design and content of the tool. We developed a semi-structured interview guide (Additional File 1) to elicit perceptions of the intervention’s relative advantage and complexity as informed by the Consolidated Framework for Implementation Research [30]. All interviews were conducted virtually between May and August 2021 and recorded through Zoom; interview recordings and notes were summarized and deidentified by LP.

We conducted a rapid qualitative analysis [31, 32] of the detailed summaries to identify main themes from the usability interviews for future refinement of the tool (MCR, LP, MEG). Subject domains from the interview guide were developed into a Microsoft Word template to organize data extracted from interview summaries. We validated the extraction template using two participant interviews; minor revisions were made to clarify broad subject domains and differences in extraction were resolved through consensus (LP and MEG). LP extracted data from the remaining interviews and synthesized results by participant and domain from the extraction template in a matrix in Microsoft Excel.

STEP 5: We developed an adoption and implementation plan for the intervention. This included identifying the intervention implementers, the outcomes they would need to achieve for intervention use, and the specific objectives and implementation strategies needed to attain and sustain those outcomes. Essentially, we repeated Steps 1–4 with the implementation of the developed intervention in mind, rather than the design of intervention content and characteristics (MCR and SS).

STEP 6: We created an evaluation plan with input from the advisory board for both a preliminary feasibility study as well as a future, larger scale study to examine the effectiveness and implementation of our intervention (MCR, SS, and LP) [33].

## Results

Step 1. We developed our logic model of the problem (underutilization of cascade screening for LS) to address each of our program goals, which were defined as: [1] *85% of first-degree relatives of probands receive information about LS test results;* and [2] *At least six relatives (first, second and/or third degree) of each proband undergo genetic testing*. Probands refer to the first member of the family to be diagnosed with LS who serves as the index patient for the cascade screening process. These goals were derived both from the systematic review as well as expert opinion from our advisory board. Our second goal was informed by work noting that under current practice, 3.6 relatives are typically tested, leading to the identification of one new LS case (14). Thus, by testing six relatives, each intervention recipient may identify one additional family member with LS compared to the number identified on average with current cascade screening practices.

Step 2. Our behavioral outcomes and performance objectives were on the patient, family member and genetic counselor levels. We specified performance objectives for each of these outcomes. For example, patients will [1] identify at-risk relatives, and [2] identify strategies for each relative based on age, closeness and receptivity, etc. Family members must [1] gather information necessary to make an informed decision to receive genetic testing for LS, and [2] receive genetic testing for LS, etc. Finally, providers need to [1] explain why patients need to communicate risk information to relatives and [2] prepare patients for communicating with relatives, etc. (Additional File 2).

The stakeholders classified 17 determinants as both important to improving cascade screening uptake and changeable based on their expert opinions (Table 1). These determinants were prioritized and ultimately grouped into five broader categories: knowledge, skills, self-efficacy, perceived barriers, and outcome expectations.

**Table 1:** Determinants for cascade screening among individuals diagnosed with LS

Level	Determinant	Identified in Qualitative Interviews	Identified in Systematic Review?	Changeability Score Average (Standard Deviation; Range)	Prioritized?
Patient	Beliefs about the relative priority of cascade screening among relatives	Y	Y	2.75 (0.5; 1)	Y
	Fear of genetic discrimination	Y	Y	2.6	Y
	Belief that cascade screening is of low relative priority	Y	Y	2.6 (0.55; 1)	Y
	Low perceived susceptibility of relatives	Y	Y	2.6 (0.55; 1)	Y
	Fear and avoidance of LS diagnosis	Y	Y	2.4 (0.55; 1)	Y
	Low knowledge about LS required for sharing information	Y	*	2.4 (0.55; 1)	Y
	Low outcomes expectancies for managing LS if tested positive	Y	N	2.2 (0.84; 2)	Y
	Guilt or stigma around uncovering a positive result	Y	Y	2.2 (0.45; 1)	Y
	Perceived costs as a barrier to testing	Y	Y	2 (0.71; 2)	Y
Provider	Low provider knowledge about LS and cascade testing	Y	Y	2.3 (0.97; 2)	Y
	Limited provider knowledge about HIPAA-allowed processes for provider to directly contact relatives/assist in cascade screening	Y	N	2.1 (0.89; 2)	Y
	Lack of provider skills to provide cascade screening	Y	Y	2 (1; 2)	Y
Family	Complex family dynamics	Y	Y	1.5 (0.5; 1)	Y
Organizational	Lack of non-English resources	Y	N	2.4 (0.89; 2)	Y
	Lack of time for provider to follow up with patients about cascade screening	Y	N	2 (0.71; 2)	Y
	Belief that it is of low relative priority in clinic	Y	N	2 (0.82; 2)	Y
Community	Limited access to genetic services (e.g., travel time, distance)	Y	Y	2.2 (0.84; 2)	Y
Policy	Lack of coverage for genetic testing and/or counseling	Y	Y	1.4 (0.89; 2)	N
	Low access due to high test/follow-up costs	Y	N	1.3 (0.45; 1)	N

Least endorsed determinants on the basis of changeability and importance included lack of coverage for genetic testing and low access due to high test costs and follow up costs. Of note, these three determinants were on the policy and organizational levels. Determinants were mapped to broader constructs from the social cognitive theory and the health belief model, such that our final determinants included knowledge, skills, self-efficacy, outcome expectancy, and perceived barriers. Next, we (MCR and SS) mapped these determinants to our change objectives and developed our logic model of change which we compared to the logic model developed externally using the behavior change wheel. Minor adjustments to the content of key messages were made based on this comparison in Step 4, including adding more information about surveillance and management strategies for LS and bolstering guided practice for communication of LS results with family members. Using these findings from Step 2, we developed a logic model of change (Additional File 3).

Step 3. Because our needs assessment identified protecting family health as a key motivator for patients to engage in cascade screening, program themes were selected to highlight the potential impact of cascade screening on family health. Images of families were selected for the cover of *Let’s Talk*. Our determinants and change objectives were aligned with theory- and evidence-based methods including: information chunking, guided practice, goal setting, setting graded tasks, gain-framing, networks, and planning coping responses (Additional File 2). We then considered how to operationalize these methods by selecting design applications. These applications were developed to fit within a paper-based workbook (and an editable pdf) and included educational materials (e.g., fact sheets), goal setting activities, guided practice prompts, and motivational messaging for patients (Table 2), as well as educational materials for genetic counselors.

**Table 2:** Summary methods and applications for *Let’s Talk* workbook

Determinant	Patient & Family		Genetic Counselor	
	**Methods**	**Applications**	**Methods**	**Applications**
Knowledge	Information chunking	Educational material	Educational meeting	Workshop
Skill	Goal setting; guided practice	SMART goal setting; guided prompts	Skills training	FAQ with communication prompts
Self-efficacy	Guided practice; graded tasks	Guided prompts; order prompts easiest to hardest	Guided practice	Role play
Perceived barrier	Planning coping responses	Guided prompts	Plan coping responses	Role play
Outcomes expectation	Gain framing; anticipated regret	Motivational messaging	Gain framing	Motivational messaging

Step 4. After review of the draft workbook by the advisory board, we made changes to the intervention language and added new supplementary resources. Images used in the workbook were changed from graphic icons to images of families. We edited the workbook iteratively until a final workbook in an editable pdf format as well as a print version were finalized.(see Additional File 4).

The ten participants in the usability study reviewed the editable pdf format of the workbook. The results of the rapid qualitative analysis found the intervention was acceptable to patients with Lynch syndrome in terms of complexity and relative advantage to other resources (Table 3).

**Table 3:** Preliminary Usability testing Results for *Let’s Talk* workbook

Design and Format		# of Participants (*n* = 10)
**Strengths**	Reported the workbook was easy to use	8
	Liked how the information was presented in simple language and broken down into smaller points	6
	Appreciated the ample blank spaces available for notetaking	5
**Suggestions**	A web-based version of the workbook would be acceptable	10
	Reformat Prompt 1 (Identify family members) to maximize use of space and capture additional family details	6
	Expressed interest in an app version of the workbook with additional features for Lynch syndrome care management	5
Content		
**Strengths**	Liked the Supplemental Resources as a place for more information to meet different needs and support for communication	7
	Liked how common concerns raised by family members were addressed with strategies for responding	5
**Suggestions**	Expand information on Lynch syndrome-related cancers beyond colorectal and endometrial cancer and specific cancer risks associated with genetic variants	7
	Add information for cascade screening in children including strategies for communication and a reminder that testing is recommended at 18 years old	5
	Note the difficulty of establishing life insurance following a diagnosis	4

All participants noted they would recommend the workbook as a tool for family communication to individuals recently diagnosed with Lynch syndrome and 5 of the 10 participants emphasized that similar materials for guiding family communication about Lynch syndrome were not available. Eight of ten participants found the workbook easy to use. Participants had several suggestions for enhancing the information on Lynch syndrome and exercises in the workbook including expanding information on cancer types and risks associated with Lynch syndrome, the challenges of establishing life insurance, and techniques for communicating with children.

Finally, the usability study participants emphasized the importance of flexibility in intervention format to meet the preferences of diverse users. Eight participants stated they would prefer to use a print workbook, but all participants noted a web-based version of the workbook would be acceptable under certain circumstances such as enhanced data security and maintaining options to print content. A workbook app was less popular because participants viewed family communication as a temporary phase; however, five participants expressed interest in an app-based intervention if communication guidance were offered with additional services for managing LS, such as a place to find up-to-date personalized screening recommendations and coordinate doctor appointments.

Step 5. Stakeholders agreed that genetic counselors were best suited to support cascade screening. As such, we identified genetic counselors as potential implementers of *Let’s Talk*. We also acknowledged that genetic counseling workforce shortages and barriers to the access of genetic counselors are prevalent, so we planned for *Let’s Talk* to be introduced by a genetic counselor but completed at home by patients and relatives. We developed outcomes, performance and change objectives for the implementation of *Let’s Talk* (see Table 4). Educational and training materials for program implementers were developed including a PowerPoint presentation with activities that can be used in an educational meeting with genetic counselors and research staff or provider champions (Additional File 5).

**Table 4:** Implementation outcomes, determinants, and strategies for *Let’s Talk*

Decision Maker	Implementation Outcome	Determinants of Change	Implementation Strategy
Organizational		Knowledge/Awareness Attitudes Outcome Expectations Skill and Efficacy	
Medical Director or Program Coordinator	Support the use of *Let’s Talk* in genetics clinics through training and resources		Develop an implementation blueprint for a clinic that adopts *Let’s Talk*
Provider			
Genetic Counselor	Provide *Let’s Talk* to probands and support family member communication		Conduct educational meetings for the delivery of *Let’s Talk* featuring a toolkit for genetic counselors Provide ongoing consultation during dissemination
Patient			
Probands	Complete *Let’s Talk* activities		Obtain and use patient and family feedback to refine delivery of *Let’s Talk*
Family Members	Seek genetic testing for LS and complete *Let’s Talk* activities		

Step 6. We developed an evaluation plan with key indicators and measures. We developed these key indicators and measures for preliminary feasibility studies as well as questions, measures and design for a larger evaluation when broader intervention testing will be conducted (see Table 5).

**Table 5:** Evaluation outcomes for *Let’s Talk* workbook

Indicator	Description	Measure
*Effect Outcomes*		
Reach of At-Risk Family Members (Patients Only)	What percentage of the family members (listed as at-risk by probands) were contacted by probands regarding genetic testing for LS?	Quantitative self-report survey
Genetic Counselor Contact (Patients Only)	How many of those family members contacted genetic counselor(s) to pursue genetic testing?	Quantitative self-report survey
Genetic Testing Uptake (Patients Only)	How many of those family members received genetic testing?	Quantitative self-report survey
*Process Outcomes*		
Acceptability	What is the satisfaction with the intervention?	5 to 6 Question Likert Scale Assessment^1^
Demand	What is the expressed intention to use the intervention? What are the perceived negative and positive effects of using the intervention?	5 Question Likert Scale Assessment for Self-Efficacy^2^, Qualitative interview analysis
Implementation	What were the resources (time, cost, etc.) required to implement the intervention?	Quantitative self-report survey^3^, Qualitative interview analysis
Practicality	What factors affect ease, speed, efficiency or quality of implementation of the intervention?	Qualitative interview analysis
Integration (Genetic Counselors Only)	What is the perceived sustainability of the intervention? What is the perceived fit with the existing infrastructure in clinical practice	Qualitative interview analysis

## Discussion

We used intervention mapping to develop a workbook named *Let’s Talk* that is responsive to barriers and facilitators described by key stakeholders in cascade screening for LS. Given competing demands among genetics providers during the return of genetic results, we developed an intervention that can be completed by patients and relatives at home. The intervention addresses gaps in knowledge, skills, self-efficacy, outcome expectancy and other perceived barriers (e.g., relatives’ reactions to learning an LS diagnosis). Implementation strategies include educational materials and a training module on using the tool for providers delivering LS diagnoses to patients. If effective, this intervention will increase the percentage of patients discussing their LS diagnosis with relatives as well as the number of relatives whom they contact and in turn increase the number of relatives who receive genetic testing.

This study adds to a limited body of research on interventions to improve cascade screening among families affected by Lynch Syndrome. A recent systematic review of interventions for family communication about genetic testing for hereditary breast and ovarian cancer and Lynch syndrome found only three studies testing interventions specifically targeting Lynch syndrome communication (25). These interventions utilized different methods for intervention development but, similar to *Let’s Talk,* focused on improving genetic testing uptake and family communication through patient education with written educational materials and provider-led counseling (25). The KinFact intervention was developed to improve family communication around hereditary breast and colorectal cancer and shares several features with *Let’s Talk* despite different intervention development methods. Creation of the KinFact intervention was based on multiple health behavior and communication theories including the Management of Meaning Theory and the Health Belief Model. Like *Let’s Talk*, the KinFact intervention featured education about cancer risk and prevention options, motivational messaging, and prompts for identifying relatives and planning when and how to talk about family cancer risk [34]. The KinFact intervention and *Let’s Talk* differ in their approach to planning family communication through the level of interaction provided in their prompts. The KinFact intervention prompts list topics for the intervention user to consider when talking to family members, while *Let’s Talk* uses guided activities for the user to complete for organizing personalized communication with each relative. *Let’s Talk* also addresses multilevel barriers to cascade screening such as limited access to genetics services providers and insurance restrictions through informational resources in addition to considering the patient-level barriers addressed in the KinFact intervention design.

Other studies have used intervention mapping to develop interventions to improve care in many therapeutic areas such as cancer screening, HIV and STI prevention, influenza prevention, cancer quality of life, and secondary stroke prevention [35, 36]. Prior studies have similarly found intervention mapping to be a useful method for designing health promotion interventions and identifying barriers and facilitators to the use of evidence-based practices (36, 37). Our use of intervention mapping expands the literature on this method by integrating implementation science frameworks to address multilevel barriers and facilitators for cascade screening. In particular, *Let’s Talk* addresses two limitations of existing interventions for Lynch Syndrome cascade screening, specifically the presence of multilevel barriers to cascade screening and limited use of evidence-based change methods. In the field of cancer prevention and control, the design of interventions with intervention mapping has primarily been guided by individual-level frameworks that do not address behavior change at other environmental levels (37). The content and design of *Let’s Talk* address patient, provider, family member, organizational, and system-level barriers to cascade screening. In particular, *Let’s Talk* includes information for overcoming system-level barriers to cascade screening including information on legal protections for employment and insurance and resources for accessing genetic counseling services. Although we did not directly address policy and organizational level determinants due to low expectations of changeability among stakeholders, we did provide informational resources addressing some of these barriers, such as information about legal protections for employment and insurance. Additionally, *Let’s Talk* incorporates multiple evidence-based methods in a single intervention to promote behavior change for communication at the patient, family member, and provider levels. *Let’s Talk* incorporates additional evidence-based methods including goal setting and guided prompts to help patients plan for active communication about cascade screening with their relatives.

The use of intervention mapping increased the potential effectiveness of *Let’s Talk* to promote cascade screening for LS in two major ways. First, the systematic intervention mapping process identified theory-based methods and strategies to deliver content addressing known barriers to cascade screening. Second, the approach considered the unique context of communication around family testing for LS that will influence the implementation and effectiveness of the intervention in its design. Specifically, we aligned the characteristics of *Let’s Talk* with the needs of key stakeholders in cascade screening to develop a multi-level intervention equipped for a wider impact in LS care. The role of the advisory board ensured the perspectives of stakeholders in LS care were accounted for in the selection of intervention objectives, methods, strategies, and implementation during intervention development.

The preliminary results on intervention acceptability and ease of use from the patient perspective are promising for future benefit in clinical practice. However, there are limitations to the intervention design and the usability study findings that must be considered. First, in Step 2 of the intervention mapping process, only a small group of five stakeholders rated the changeability of key determinants of LS cascade screening. While the stakeholders represented multiple perspectives in LS care (i.e. patients, clinicians, and researchers), changeability scores may not be generalizable. These scores along with salience of these determinants in the literature and qualitative needs assessment led our advisory panel to prioritize all determinants except policy-level barriers to testing affordability and coverage in the design of Let’s Talk. We feel this choice by stakeholders recognizes the need for immediate support for LS patients and providers in navigating the complex process of cascade testing in the US healthcare system. This also highlights the need for additional research to develop policy-level solutions to existing barriers. We also recognize that other patients, genetic counselors, physicians, and implementation scientists may have prioritized different or fewer determinants of cascade screening for LS and a more sensitive measure of determinant priority than low [1] to high [3] may be helpful to highlight the most critical barriers to cascade screening for immediate research and policymaking. Second, we did not include genetic counselors in the usability study, so the perspective of providers who will implement *Let’s Talk* with patients in practice remains to be evaluated. Additionally, the usability study was conducted with a small sample of 10 LS patients and demographic characteristics were not collected. The sample may not represent the average LS patient in technical proficiency and LS engagement as all participants were recruited through the Lynch Syndrome International Facebook page. Further research is underway to understand the acceptability of the *Let’s Talk* for diverse multi-level users of various cultural, educational, and clinical backgrounds.

Next steps include further testing the tool using the implementation and evaluation plans developed. If effective, we would transform the paper-based version to a web-based tool, as recommended by several key stakeholders and usability testing participants in step 4. To this end, intervention mapping techniques could be leveraged to adapt the intervention to an online platform. Benefits of an online platform would include easier dissemination and flexible accessibility for using the tool, real time messaging and downloading of materials such as genetic test results between patients, relatives and provider, and continuous reminders for patient engagement. Further, this intervention can be adapted to include additional hereditary conditions, such as hereditary breast and ovarian cancer and familial hypercholesterolemia.

## Conclusions

We used intervention mapping, a systematic 6-step process for stakeholder-driven intervention development, to create *Let’s Talk*, an educational workbook for patients with Lynch syndrome to improve the uptake of cascade screening. We mapped performance and change objectives, methods, and strategies to the determinants of LS cascade screening at the individual level to the policy levels. The final form of *Let’s Talk* features educational content, goal setting and planning prompts, motivational messaging, and supplemental resources for patients delivered through evidence-based methods including information chunking, guided practice, and gain-framing. The results of intervention pretesting suggest *Let’s Talk* will be a useful resource for family communication around LS, although a web-based format and additional content about cancer risks and communication with minors could improve the flexibility of the tool to fit patient- and family-specific contexts for use. The strengths of *Let’s Talk* include the use of evidence-based methods, key stakeholder input, and pretesting in patients in intervention design while *Let’s Talk* development was limited by a small sample for pretesting. Future research plans include transforming *Let’s Talk* into a web-based tool and assessing the intervention in clinical practice with patients and genetic counselors. Other researchers should consider the use of intervention mapping as an evidence-based technique to develop stakeholder-centered interventions for addressing barriers to care for other unique populations including those with other hereditary conditions.

## Supplementary Information

**Additional file 1.** Interview Guide; This file contains the interview questionnaire used with patient during usability testing of the intervention.

**Additional file 2.** Evidence-Based Method Mapping; This file shows the evidence-based method selected to address each change objective and determinant.

**Additional file 3.** Logic Model; The logic model shows a mapping of the change process for patients, relatives, and providers.

**Additional file 4.** Let’s Talk Workbook; This document contains the latest version of *Let’s Talk*, the intervention designed through this intervention mapping process.

**Additional file 5.** Let’s Talk Provider Manual; This PowerPoint presentation contains activities that can be used in an educational meeting with genetic counselors and research staff or provider champions to introduce *Let’s Talk*.

## Data Availability

The datasets used and analyzed during this study are available in the additional files or from the corresponding author on reasonable request.

## Abbreviation

LS: Lynch Syndrome
