# Supplementary material for: Development and initial testing of a multi-stakeholder intervention for Lynch syndrome cascade screening: an intervention mapping approach
Source: BMC Health Serv Res. 2022 Nov 24;22:1411. doi: 10.1186/s12913-022-08732-6 (PMC9694070; doi:10.1186/s12913-022-08732-6)
Supplement: Supplementary file 1 — Additional file 1. Interview Guide; This file contains the interview questionnaire used with patient during usability testing of the intervention. [file 12913_2022_8732_MOESM1_ESM.docx]

Note: This is a semi-structured interview, and exact questions asked will vary based on the conversation.

Let’s Talk Usability Study Interview Guide

Introduction: Hello, thanks for joining us. My name is < interviewer name> and I’m a member of the research team working with <principal investigator name> on this study and I’ll be leading your interview today. *Optional: With me, we have <other interviewer name> who will be assisting in the interview and taking notes.* We’ll be talking about your thoughts on Let’s Talk, the educational workbook we’re developing for patients with Lynch Syndrome. We’re looking to hear your opinion on the strengths and weaknesses of the workbook design. We are interested in all kinds of feedback – positive and negative – to help us improve the workbook for other patients. Negative feedback won’t hurt my(our) feelings; we appreciate your openness and honesty in providing feedback.

The interview should last approximately 45 to 60 minutes. You can skip any questions you do not want to answer, and you can end the interview at any time. Do you have any questions before we get started?

Are you okay to start the interview now? [If yes, continue]. Are you okay with starting the recording now [If yes, start recording].

*Prompts: Can you give examples? Is there anything else? Is that something that’s important to you? Some previous things you mentioned are X, Y, and Z? What do you mean by X, can you explain this further? So it sounds like X is important to you or you think X?

Theme #1 Relative Advantage

1. Now that you’ve gone over the workbook – what did you think about it? [PAUSE FOR ANSWER]
   1. What did you like about the workbook?
   2. What did you dislike about the workbook?
2. How does the workbook compare to other materials you may have seen on family testing for Lynch Syndrome? [PAUSE FOR ANSWER]
   1. In what ways is it more helpful?
   2. In what ways is it less helpful?
3. In what ways do you think this workbook helps a patient have conversations about Lynch Syndrome?
4. What suggestions do you have for improving the workbook? [PAUSE FOR ANSWER]
   1. How could the information be improved or changed?
   2. How could the exercises be improved or changed?

Theme #2: Practicality

Introduction: Now I’m interested in learning more about how easy or difficult it was to use the workbook, as well as any suggestions you have for improving its design.

1. How easy or difficult was it to use the workbook? [PAUSE FOR ANSWER]
   1. What was easy?/what makes it easy?
   2. What was difficult?/What makes it difficult?
2. Were there any part of the workbook you found difficult to understand or complete? [PAUSE FOR ANSWER] Please explain.
3. How do you feel about the length of the workbook? [PAUSE FOR ANSWER] (too long, too short, just right?)
   1. How do you feel about the length of the exercises?
4. Was the organization of the exercises logical? [PAUSE FOR ANSWER]
   1. Would you change the order of the workbook sections?
5. In what ways, if any, can the workbook be improved so that it is easier to use?
   1. How can the exercises be improved so they are easier to understand?
6. What would be your preferred format for using the workbook (paper, web-based, app?) Why?
   1. How do you feel about a web-based/browser version? What concerns do you have?
   2. How do you feel about a mobile app version? What concern do you have? How do you think those concerns could be addressed?

Theme #3: Demand

Introduction: As concluding questions...

1. Would you recommend the workbook to people recently diagnosed with Lynch Syndrome? Why or why not?
2. Is there anything else you’d like to share before we wrap up today?

Conclusion:

Thank you so much for taking your time to review this workbook and for speaking with us today. We appreciate hearing all of your feedback on the workbook design and ways we can improve the workbook to make it a better resource for individuals with Lynch Syndrome.
