# Supplementary material for: Development and initial testing of a multi-stakeholder intervention for Lynch syndrome cascade screening: an intervention mapping approach
Source: BMC Health Serv Res. 2022 Nov 24;22:1411. doi: 10.1186/s12913-022-08732-6 (PMC9694070; doi:10.1186/s12913-022-08732-6)
Supplement: Supplementary file 3 — Additional file 3. Logic Model; The logic model shows a mapping of the change process for patients, relatives, and providers. [file 12913_2022_8732_MOESM3_ESM.pptx]

## Slide 1
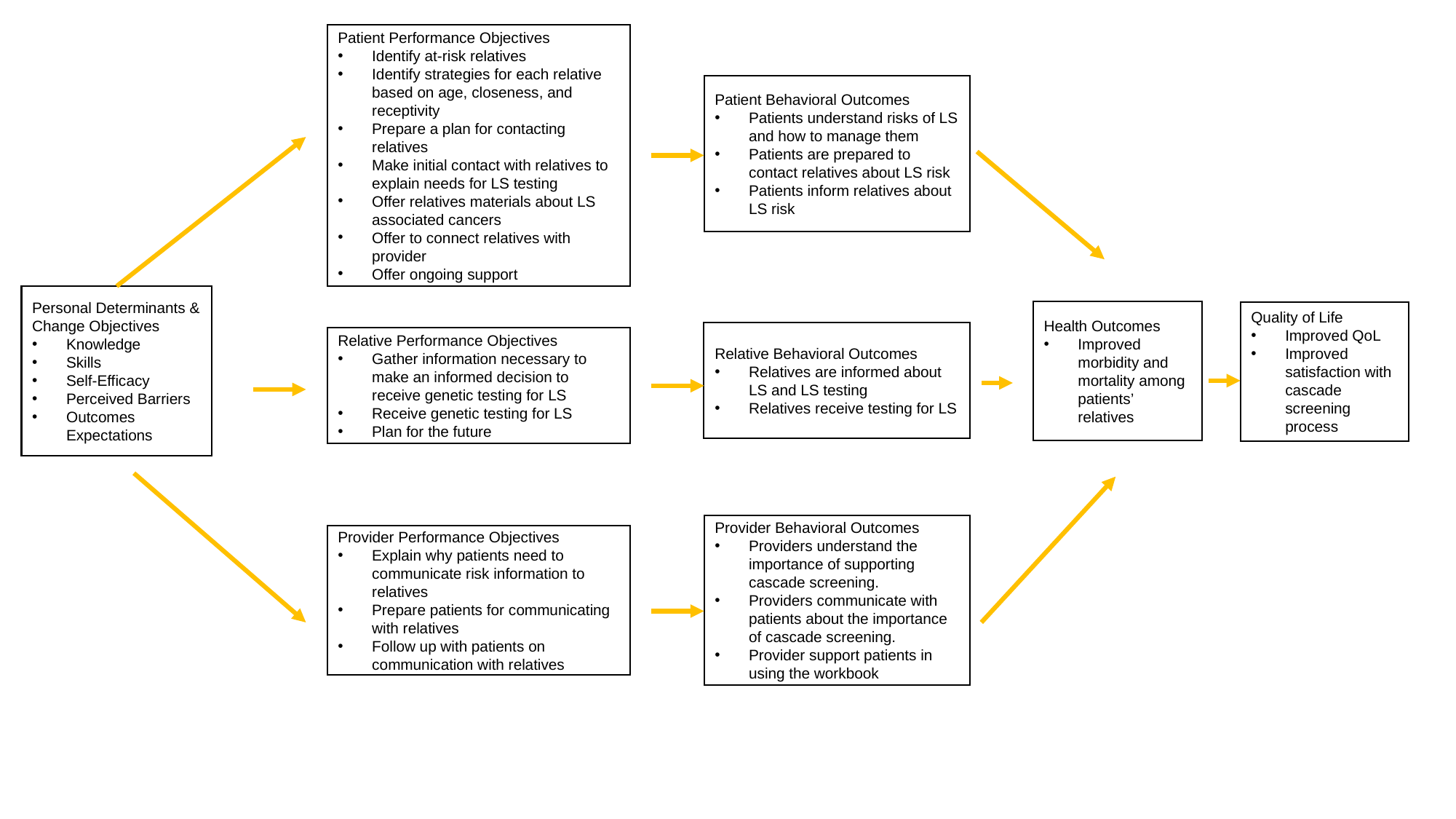

Patient Performance Objectives
Identify at-risk relatives
Identify strategies for each relative based on age, closeness, and receptivity
Prepare a plan for contacting relatives
Make initial contact with relatives to explain needs for LS testing
Offer relatives materials about LS associated cancers
Offer to connect relatives with provider
Offer ongoing support
Patient Behavioral Outcomes
Patients understand risks of LS and how to manage them
Patients are prepared to contact relatives about LS risk
Patients inform relatives about LS risk
Personal Determinants & Change Objectives
Knowledge
Skills
Self-Efficacy
Perceived Barriers
Outcomes Expectations
Health Outcomes
Improved morbidity and mortality among patients’ relatives
Quality of Life
Improved QoL
Improved satisfaction with cascade screening process
Relative Behavioral Outcomes
Relatives are informed about LS and LS testing
Relatives receive testing for LS
Relative Performance Objectives
Gather information necessary to make an informed decision to receive genetic testing for LS
Receive genetic testing for LS
Plan for the future
Provider Behavioral Outcomes
Providers understand the importance of supporting cascade screening.
Providers communicate with patients about the importance of cascade screening.
Provider support patients in using the workbook
Provider Performance Objectives
Explain why patients need to communicate risk information to relatives
Prepare patients for communicating with relatives
Follow up with patients on communication with relatives
