# Supplementary material for: Development and initial testing of a multi-stakeholder intervention for Lynch syndrome cascade screening: an intervention mapping approach
Source: BMC Health Serv Res. 2022 Nov 24;22:1411. doi: 10.1186/s12913-022-08732-6 (PMC9694070; doi:10.1186/s12913-022-08732-6)
Supplement: Supplementary file 4 — Additional file 4. Let’s Talk Workbook; This document contains the latest version of Let’s Talk, the intervention designed through this intervention mapping process. [file 12913_2022_8732_MOESM4_ESM.pdf]

Let's Talk

about Lynch syndrome and your family

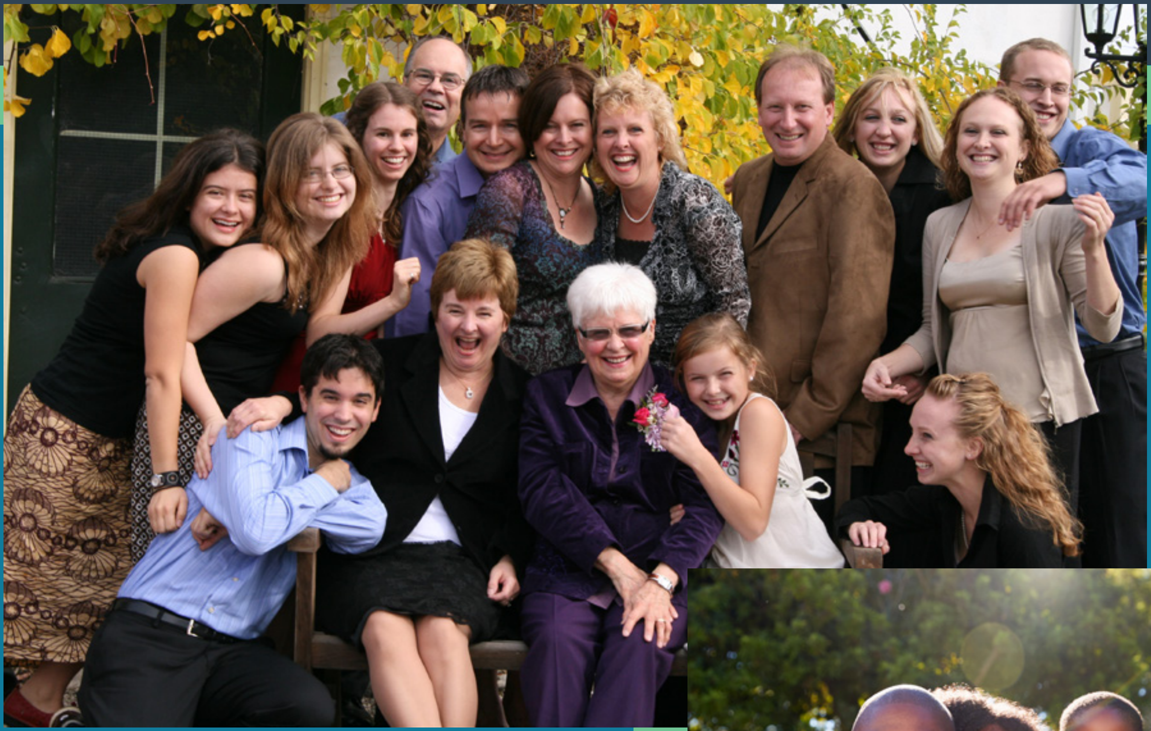

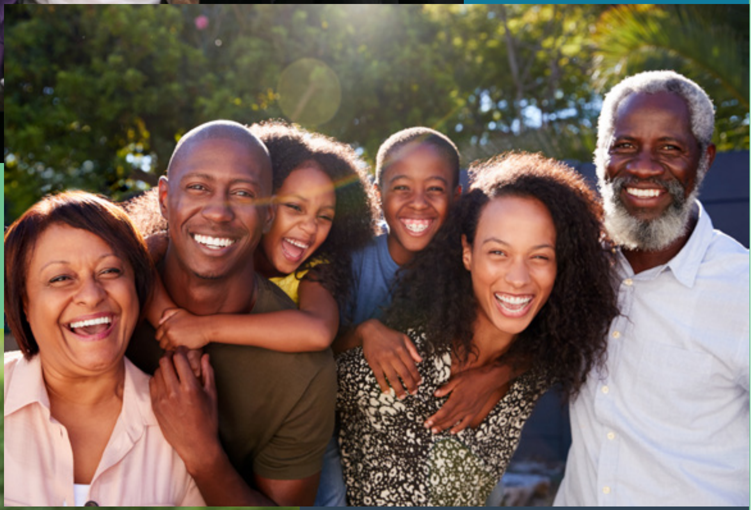

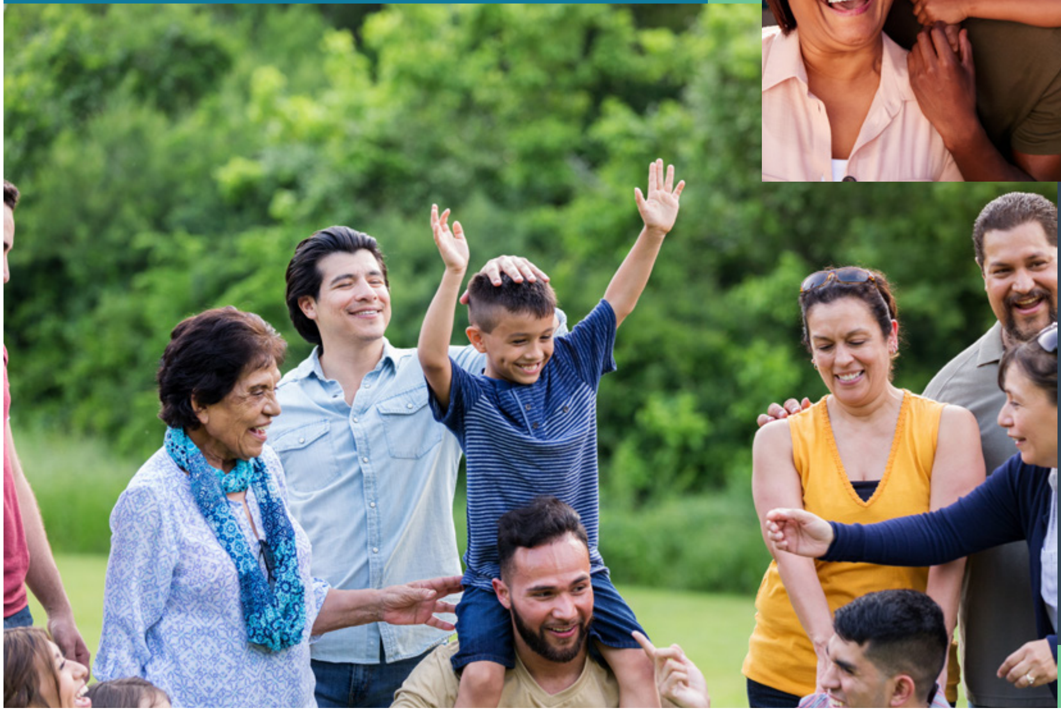

Contents

Welcome 1

Why should I tell my family members about Lynch syndrome? 3

Who's at risk? Who do I need to reach out to? 4

What should I share with my family members about Lynch syndrome? 8

How should I tell my family members about Lynch syndrome? 15

List of Terms 22

Cancer Prevention Measures 24

Frequently Asked Questions 25

Resources 26

Notes 28

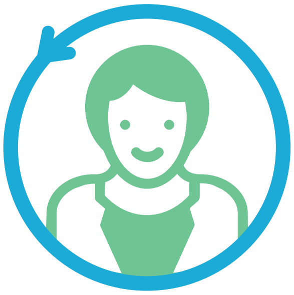

Lynch syndrome affects you and your family.

This workbook is meant to help you find ways to talk with people in your family about LS.

Let's Talk

about Lynch syndrome and your family

Welcome to Let's Talk about Lynch syndrome and your family.

This workbook will help you develop a plan to share information about Lynch Syndrome (LS) with your family members. This process can be hard. Take your time, and don't feel like you have to finish this workbook in one day.

Sharing this information can be lifesaving for your family members.

In this workbook, we'll work through this process step-by-step. We'll try to answer these questions:

1. Why should I tell my family members about LS?
2. Who's at risk? Who do I need to reach out to?
3. What should I share with my family members about LS?
4. How should I tell my family members about LS?

Reach out to your genetic counselor, or another trusted provider, for support if you need it.

Provider ____

Phone ____

Email ____

Would you like to talk to others with LS? Discuss your experiences about sharing information with your family members (see the links to online groups in the Resources section on page 26).

What is LS?

LS is a syndrome that means you have an increased risk of getting some types of cancer. LS can be passed down from one generation to the next.

The most common cancers that those with LS may develop are:

- colorectal
- endometrial or uterine (women only)

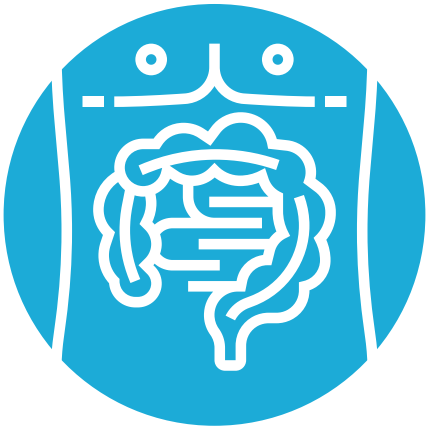

colorectal

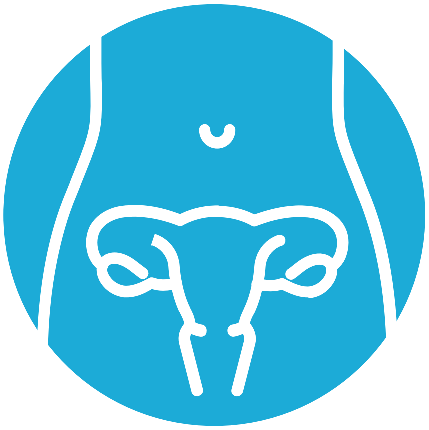

endometrial or uterine

What do I need to know if I have LS?

Having LS doesn't mean that you already have cancer. It does mean that you have a higher chance of getting colorectal or endometrial cancer than the average person.

You should follow the advice of your medical team to help reduce your chances of cancer.

- Get screened for cancer early and often to catch signs of cancer.
- Talk to your doctor about whether it may be good for you to get some types of surgery to prevent cancer.

Your family members might also have LS. It's important for them to learn about it, too.

Why should I tell my family members about LS?

It's important to share that you have LS with your family members so they can get tested for LS, too. If they do have LS, they can talk to their doctor about what to do to reduce their risk of cancer.

Share this lifesaving information with your family members! For your relatives, knowing whether they have LS gives them and their doctors the chance to make important decisions about their health.

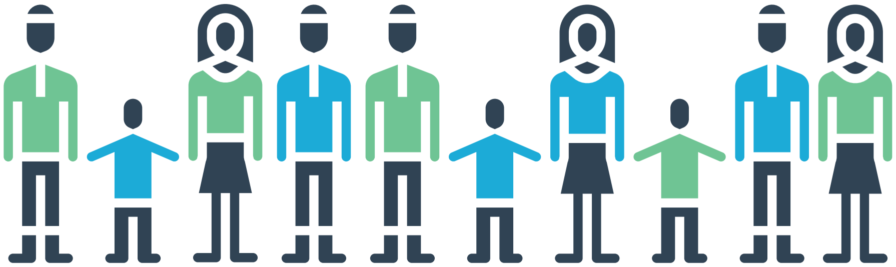

Who's at risk? Who do I need to reach out to?

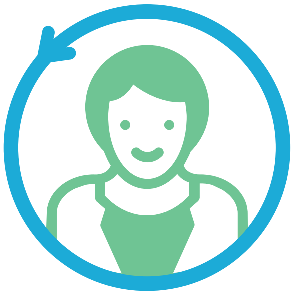

Your parents, children, brothers, and sisters have a 50% chance of having LS. It's important to tell these family members about LS first, so they can be tested and share their results with their first-degree relatives. If they test negative, they can receive general cancer screenings and their children do not need to receive LS testing. First-degree family members who choose not to be tested should receive increased screening for LS-related cancers.

PROMPT #1

Who do I need to reach out to?

You might not feel comfortable contacting each of your relatives. That's okay! As you fill out this section, take a moment and think about who could help you contact family members.

MOTHER

Name ____

Are you comfortable discussing LS with this relative?

◯ Yes ◯ No ◯ Not sure yet

FATHER

Name ____

Are you comfortable discussing LS with this relative?

◯ Yes ◯ No ◯ Not sure yet

BROTHERS AND SISTERS

Name ______________________________

Age ______________________________

Are you comfortable discussing LS with this relative?

○ Yes ○ No ○ Not sure yet

Name ______________________________

Age ______________________________

Are you comfortable discussing LS with this relative?

○ Yes ○ No ○ Not sure yet

Name ______________________________

Age ______________________________

Are you comfortable discussing LS with this relative?

○ Yes ○ No ○ Not sure yet

BROTHERS AND SISTERS

Name ______________________________

Age ______________________________

Are you comfortable discussing LS with this relative?

Yes   No   Not sure yet

Name ______________________________

Age ______________________________

Are you comfortable discussing LS with this relative?

Yes   No   Not sure yet

Name ______________________________

Age ______________________________

Are you comfortable discussing LS with this relative?

Yes   No   Not sure yet

CHILDREN

Name ______________________________

Age ______________________________

Are you comfortable discussing LS with this relative?

Yes   No   Not sure yet

Name ______________________________

Age ______________________________

Are you comfortable discussing LS with this relative?

Yes   No   Not sure yet

Name ______________________________

Age ______________________________

Are you comfortable discussing LS with this relative?

Yes   No   Not sure yet

What should I share with my family members about LS?

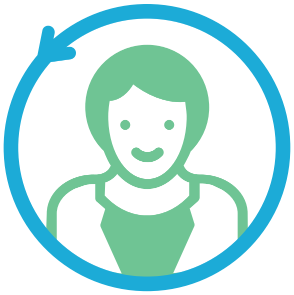

How can I help my family member find ways to learn more about LS and how to get tested?

Here are some key points to tell your family member:

Let them know what LS is and what it means.
Tell them about cancer screening and prevention (see page 24).

- People with LS have an increased risk of getting some types of cancer (primarily colorectal and endometrial).
- LS can be passed down from one generation to another, so if you have LS, you could pass LS to your children.
- Having LS doesn't mean that you already have cancer.
- Your medical team can help you take steps to prevent these cancers. Steps might be taking medication or screening earlier and more often for signs of cancer.

Give them a paper or electronic copy of your LS test result. (It's okay to mark out your name.)

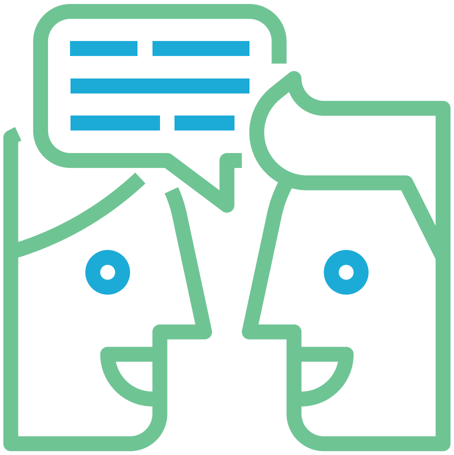

Tell them about the benefits of receiving LS testing.

- Knowing whether you have LS gives you the chance to talk to your doctors about steps to prevent LS cancers.
- If you have LS, sharing information with your family members will give them to chance to make important decisions about their health for preventing LS cancers. This can be lifesaving!

Talk about the most common myths about LS testing (see FAQs on page 25).

Offer the contact information of your genetic counselor or other health provider.

Family member(s) who don't live near you can find a nearby genetic counselor at www.findageneticcounselor.com. They can schedule a visit on the phone or in person.

Offer links to websites you have found helpful or that were suggested to you.

Examples:

- Lynch Syndrome International (**https://lynchcancers.com/**)
- Alive and Kickn Foundation (**https://www.aliveandkickn.org/**)

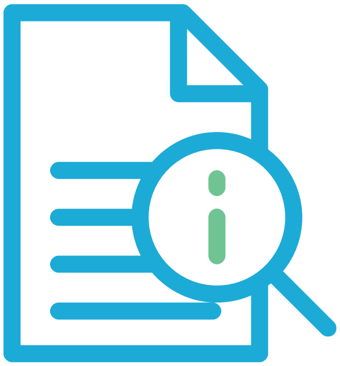

PROMPT #2

Develop a script to use when discussing LS with relatives.

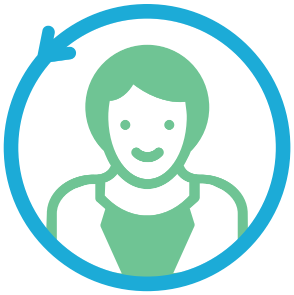

Read some examples of what you might tell your family member.

> I've recently been diagnosed with Lynch syndrome. LS is a genetic condition that increases a person's chances of developing several types of cancer, including colorectal and endometrial cancers.
>
> This condition runs in families, so it's important that you get tested, too. Getting tested for LS and knowing your LS status is beneficial. It doesn't matter if the test results are negative or positive. It's important to know.
>
> Having LS does not mean that you have cancer now. And it doesn't mean that you will definitely develop cancer in the future. It just means that you have an increased risk for cancer.
>
> If you have LS, you can take preventive steps to keep from getting cancer or to catch it early when it has the best outcomes.

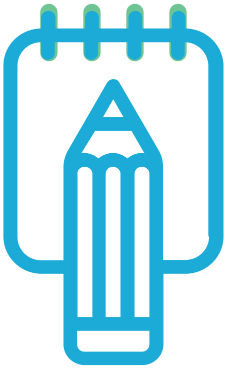

Write your own script! Some people might find it helpful to write information down. If it will help you, use the main points on pages 8 and 9 and write down a script for talking with your family members.

Your family member may express some concerns! Read some responses to some common concerns.

CONCERN	RESPONSE
**I don't like blood draws.**	Testing can be done using a saliva sample. You don't have to have your blood drawn.
**Will this affect my ability to get health insurance?**	There are laws preventing health insurers and employers from using your genetic information against you.
**I'm concerned about the cost of LS testing.**	There may be a cost for your clinic visit and a cost for the lab test. In some cases, the test may be free or there may be help to cover the costs. Talk to your doctor about your options.
**It may be hard for me to get an appointment because of my schedule.**<br>*OR*<br>**There aren't any genetic counselors in my state.**	Testing can be done in the evenings or on weekends. Phone or video may be used. When you look for a genetic counselor at **www.findageneticcounselor.com**, select one that offers counseling by phone.
**I'm afraid to know that I'm going to get cancer if there's nothing I can do about it.**	It's very important that you know that many of the LS cancers can be prevented or caught early if you follow the increased cancer screening suggested for people with LS. Knowledge is power.

Does your relative want more information on getting LS testing?

- Let them know that you can connect them with your doctor.
- Let your relative know if they might be eligible for free testing within 90 days of a positive LS test to family members.
- Share information on the testing process and groups that may be able to help as well.

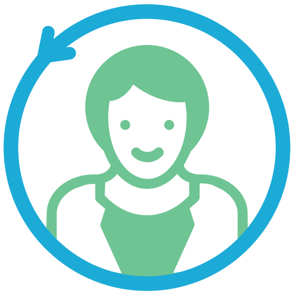

Remember: Knowledge is power!

How should I tell my family members about LS?

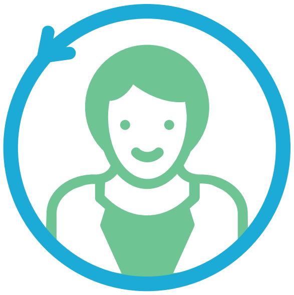

Develop a strategy for each family member. It can be hard to discuss LS with relatives. Create a plan for the "who-what-where-when-how-why." This can help you prepare for these important discussions.

PROMPT #3

Plan to reach out to each family member.

☐ **Who am I contacting?**

Name ______________________________

Age ______________________________

Are you comfortable discussing LS with this relative?

○ Yes ○ No ○ Not sure yet

If no, who might be able to help me contact this relative?

ANOTHER RELATIVE

Name ______________________________

Contact ______________________________

A FRIEND

Name ____

Contact ____

MY DOCTOR

Name ____

Contact ____

☐ **What information should I provide?**

Use the information on page 8 and your draft script on pages 11 and 12. This script can be adapted for each relative.

☐ **When would be a good time to bring up the topic?**

Find the best time to talk. For example: If your family member is young, you may want to wait to talk with them until they are at an age when they can be tested (usually 18 years old).

–

☐ **Where should I contact this family member?**

- On the phone
- Face-to-face
- Letter
- Email
- Conference call
- Other

Which will help us feel most comfortable discussing this topic?

Some methods, like a text or social media, may feel easier. But it may be harder to convey this topic and the importance of LS testing. Weigh the pros and cons of each method as you think about it. Then decide which to use.

–

☐ **How can I prepare for my relative's reactions to this news?**

- What reaction do I expect? Will my family member be upset or worried? Will they feel indifferent?
- Assure them that knowing their LS status gives them with a chance to take control of their health. Remind them they can receive the right cancer prevention measures.

☐ **Why is this process so important?**

It's important to tell my relative about LS because knowing their LS status gives them the chance to make important health decisions.

PROMPT #4

Develop goals for talking to identified relatives.

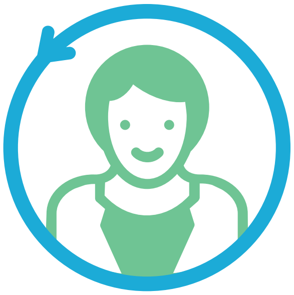

It can be helpful to create specific goals for talking with relatives.

Follow a 5-step process to create specific goals:

STEP 1 **State what action you'll take.**
Example: I'll discuss LS with all of my first-degree family members (my mom and dad, my brother, and my daughter).

STEP 2 **Define how you will track your success.**
Example: I'll aim to discuss LS with one relative each week.

STEP 3 **Make sure your goal is possible.**
Example: I'll develop a strategy for each relative below. I'll use the script I created above to guide my discussion about LS with my relatives. I'll check in with my provider to ask any questions I have, or if I need more support.

STEP 4 **Make sure that your goal is aligned with your purpose when telling first degree family members about LS testing.**
Example: Informing my family members about LS using the above methods will allow them the chance to make important health decisions.

STEP 5 **Define dates for achieving goals.**
Example: I will complete discussing LS with my first-degree family members within 8 weeks.

Remember: Sharing this information can be lifesaving!

Based on the workbook examples, this is my 5-step goal to discuss LS with my first-degree family members:

STEP 1

STEP 2

STEP 3

STEP 4

STEP 5

What should I do after I've informed my relatives?

- If you feel comfortable, follow up with family members on whether they have completed testing. You can let them know that you're able to support them. Connect them to information in this workbook.
- If your family member has LS, they should let their first-degree relatives know if they have not been tested yet.

PROMPT #5

Things to think about after you contact family members

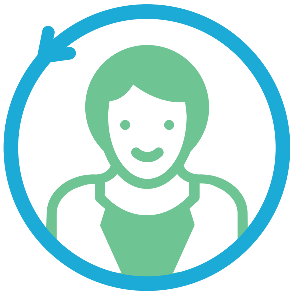

These talks or letters can be awkward, especially based on your relationship with your family member. It's normal to feel like this process didn't go completely the way you hoped.

Remember: sharing this information can be lifesaving, so give it your best! Ask for help when you need it.

As you think about this process, consider the following:

- What went well in these talks or letters?
- What could have gone better?
- What other support do you need to achieve your goals?

List of Terms

Colectomy
Partial or complete removal of the colon through surgery

Colonoscopy
A procedure used to look for abnormalities or signs of disease in the colon and rectum

Colorectal
Refers to the large bowel (colon) or the section at the end of the bowel (rectum)

DNA Mismatch Repair genes
Genes associated with LS are from a family called DNA Mismatch Repair genes. These genes include MLH1, MSH2, MSH6, PMS2, and EPCAM.

Endometrial
Refers to the lining of the uterus (womb)

First-degree Relatives
These are relatives who share approximately 50% of their DNA profile with you. This category includes biological parents, children (adult and under age 18) and siblings.

Genes
The DNA profile includes smaller elements called genes. Genes code for proteins or substances that your body needs to grow and develop properly. Your traits, like hair color and height, are caused by one or more genes. Some genes protect you from getting cancer and when these genes don't work properly, you have an increased risk of cancer.

Hereditary
This condition can run in your family, it's inherited (or passed down) from one generation to the next.

List of Terms

Hysterectomy
Removal of the uterus

LS mutations (LS pathogenic variants)
Individuals with LS have genetic mutations in the DNA Mismatch Repair genes. This means there is a change in the DNA code of a gene which causes it not to work properly. Mutations in each LS gene may be associated with a different overall risk for cancers. Thus, your medical provider should specify which gene is found to cause LS in your family.

Nucleotide
Each gene is a unique sequence of nucleotides (A, T, G, C), which are the smallest chemical unit of the DNA profile. Thus, nucleotides -> genes -> DNA profile. Each gene will have a nucleotide sequence that looks something like this: ATGGCCTTGGCC.

Oophorectomy
Partial or complete removal of one or both ovaries

Ovarian
Refers to the female organs that produce eggs

Second-degree relatives
These are relatives who share approximately 25% of their DNA profile with you. This category includes biological grandparents, grandchildren, aunts, uncles, nephews, nieces, and half-siblings.

Urinary tract
Refers to the human body's organs (like the kidneys and bladder) for removing waste

Uterine
Refers to the womb (uterus)

Measures to Prevent LS Cancers

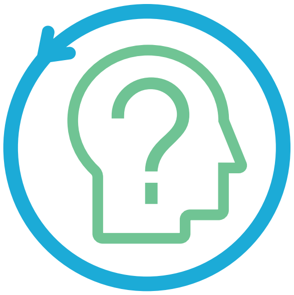

Talk with your doctor about cancer prevention options for those with Lynch syndrome:

SCREENING

- For colorectal cancer, get a colonoscopy every 1 to 2 years starting at age 20 to 25 years depending on the specific LS mutation in your family.
- For women, start screening for endometrial and ovarian cancer at age 30 to 35 years to catch signs of cancer.
- Your doctor may also suggest getting screened for stomach cancers at age 30 to 35 years, and for urinary tract cancers at age 25 to 35 years.

SURGERY

- Removal of all or part of organs may be needed if you develop tumors. Your doctor may suggest a colectomy (colorectal cancer), hysterectomy (endometrial cancer), or oophorectomy (ovarian cancer).
- Even if cancer is not present yet, surgery may be offered to prevent the growth of cancer.

Frequently Asked Questions

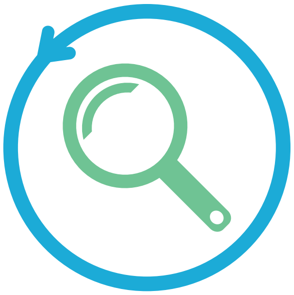

Does having LS mean that I'll develop cancer?
Having LS doesn't mean that you'll develop cancer. You can proactively take steps to to reduce your risk of cancer.

If I have LS, will that affect my career or my ability to get health insurance?
No, having LS shouldn't affect your ability to get or keep health insurance. Having LS should also not affect your ability to get or keep your job. There's a law that protects you from being treated differently by health insurance companies or employers. The law is the Genetic Information Nondiscrimination Act of 2008.

Am I the cause of LS in my family?
No! LS happens purely by chance. It's no one's fault.

If I choose to have children, can LS be passed down to my children?
Yes, there is a 50% chance of LS being passed down for each child. There are options available for people who want to have biological children but feel strongly they do not want to pass LS to biological children. You can discuss these options with your genetic counselor before you have children.

Resources

Find a genetic counselor near you or a family member.

The National Society of Genetic Counselors directory search tool
https://www.nsgc.org/page/find-a-genetic-counselor

American College of Medical Genetics and Genomics genetic clinic directory search tool
https://clinics.acmg.net/

Find a support group.

Lynch Syndrome International
Website: **https://lynchcancers.com/**
Facebook page: **https://www.facebook.com/LynchSyndromeInternational/**

AliveAndKickn: Hereditary Cancer Foundation
Website: **https://www.aliveandkickn.org/**
Facebook: **https://www.facebook.com/AliveAndKickn**

Colorectal Cancer Alliance
Website: **https://www.ccalliance.org/**
Facebook: **https://www.facebook.com/ColorectalCancerAlliance/**

Fight Lynch Syndrome
Website: **https://fightlynch.org/**
Facebook: **https://www.facebook.com/lynchsyndrome**

Fight Colorectal Cancer
Website: **https://fightcolorectalcancer.org/colorectal-cancer/lynch-syndrome/**

Facing Our Risk of Cancer Empowered (FORCE)
Website: **https://www.facingourrisk.org/**

Resources

Understand legal protections for LS patients against genetic discrimination.

Genetic Information Nondiscrimination Act of 2008 (GINA)

An introduction to GINA
https://www.genome.gov/about-genomics/policy-issues/Genetic-Discrimination

Health Insurance and Employment protections under GINA
http://www.ginahelp.org/

Patient Protection and Affordable Care Act of 2010 (ACA)

Coverage for pre-existing conditions including LS under ACA
https://www.healthcare.gov/coverage/pre-existing-conditions/

Fact sheet about guaranteed issue of insurance under ACA
https://www.kff.org/wp-content/uploads/2013/01/8327.pdf

Notes

Notes

Notes

Notes
