# Supplementary material for: Development and initial testing of a multi-stakeholder intervention for Lynch syndrome cascade screening: an intervention mapping approach
Source: BMC Health Serv Res. 2022 Nov 24;22:1411. doi: 10.1186/s12913-022-08732-6 (PMC9694070; doi:10.1186/s12913-022-08732-6)
Supplement: Supplementary file 5 — Additional file 5. Let’s Talk Provider Manual; This PowerPoint presentation contains activities that can be used in an educational meeting with genetic counselors and research staff or provider champions to introduce Let’s Talk. [file 12913_2022_8732_MOESM5_ESM.pptx]

## Slide 1
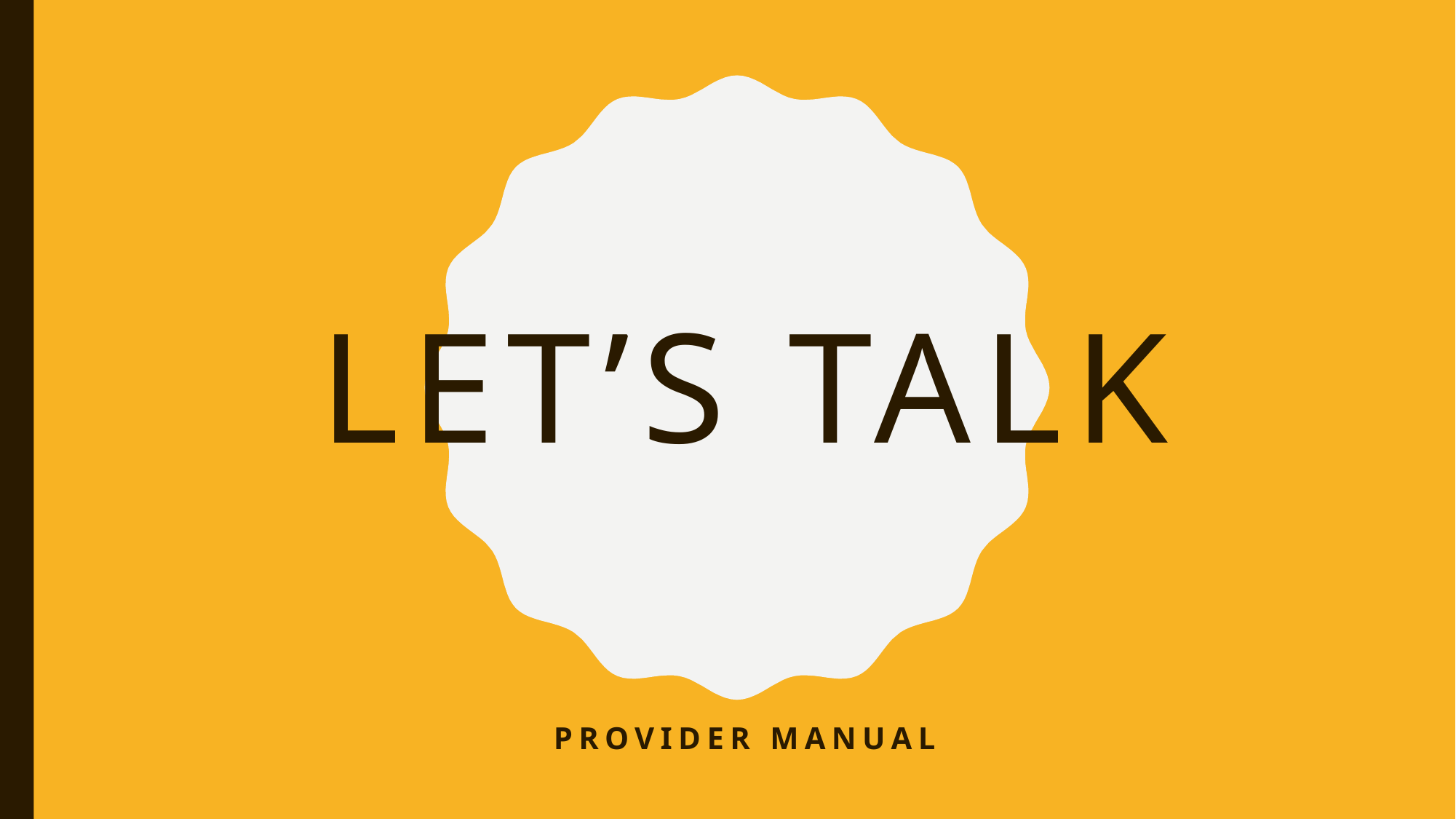

# LET’S TALK
Provider Manual

## Slide 2
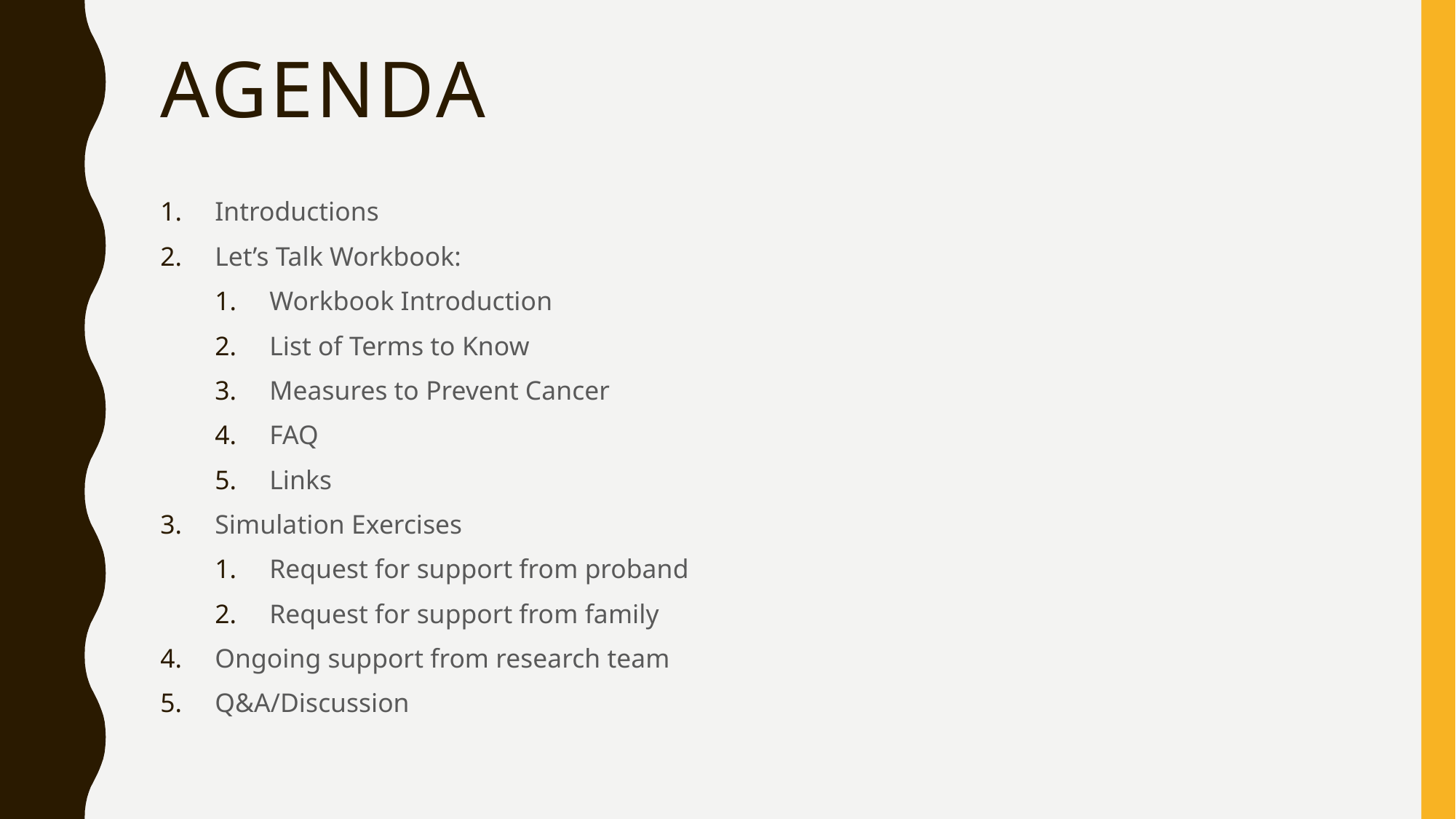

# AGENDA
Introductions
Let’s Talk Workbook:
Workbook Introduction
List of Terms to Know
Measures to Prevent Cancer
FAQ
Links
Simulation Exercises
Request for support from proband
Request for support from family
Ongoing support from research team
Q&A/Discussion

## Slide 3
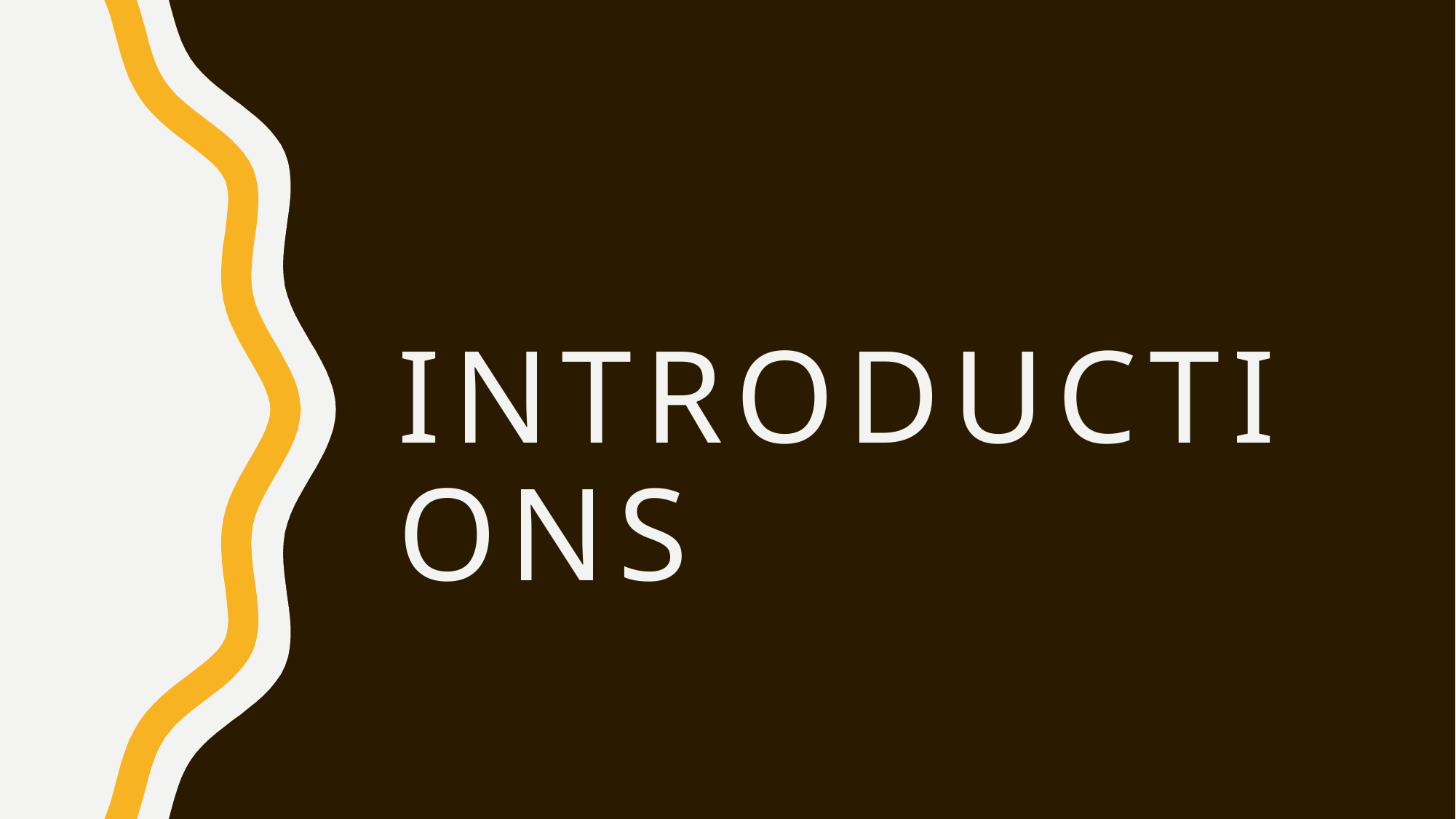

# INTRODUCTIONs

## Slide 4
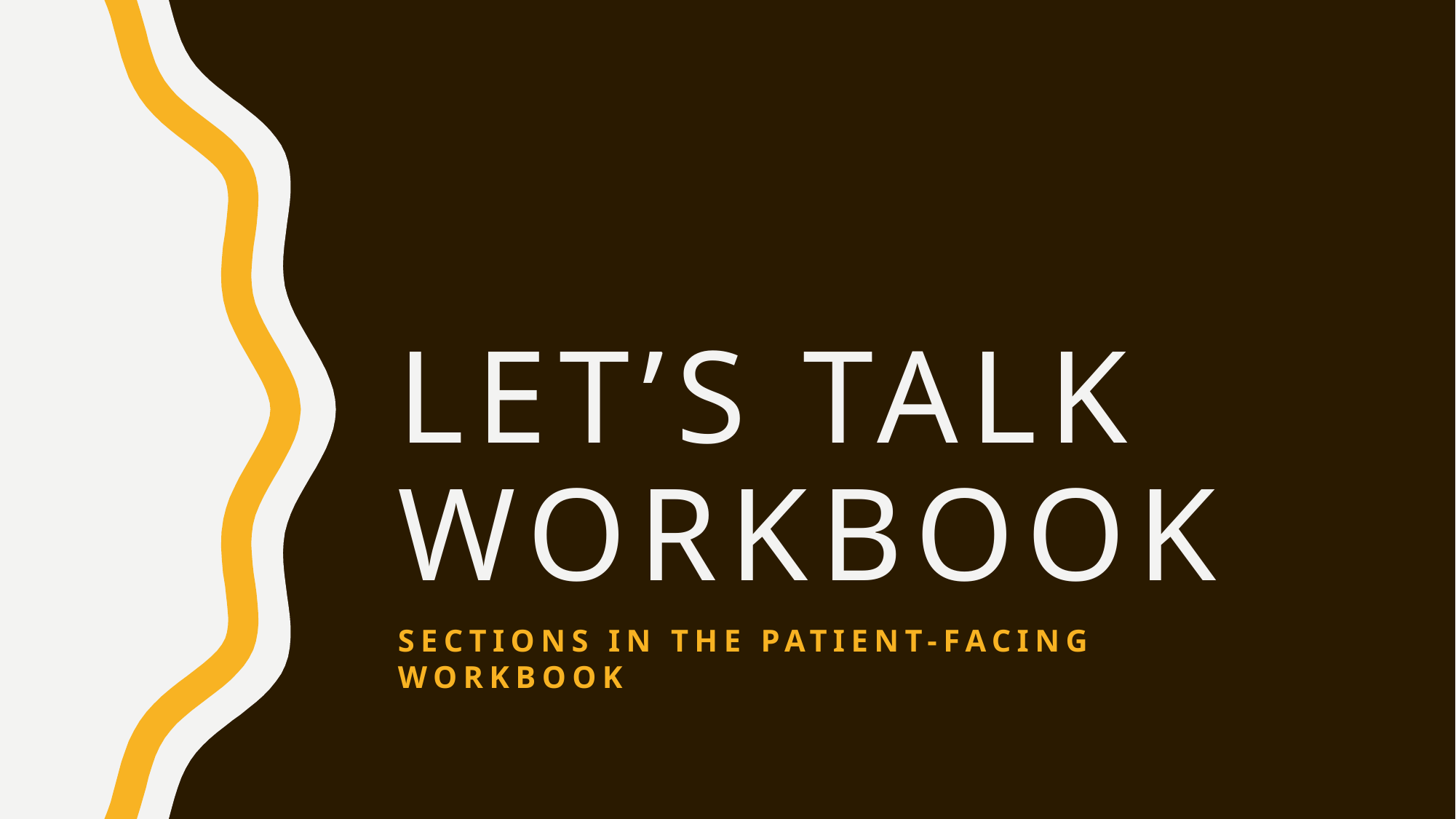

# Let’s TALK WORKBOOK
SECTIONS IN THE PATIENT-FACING WORKBOOK

## Slide 5
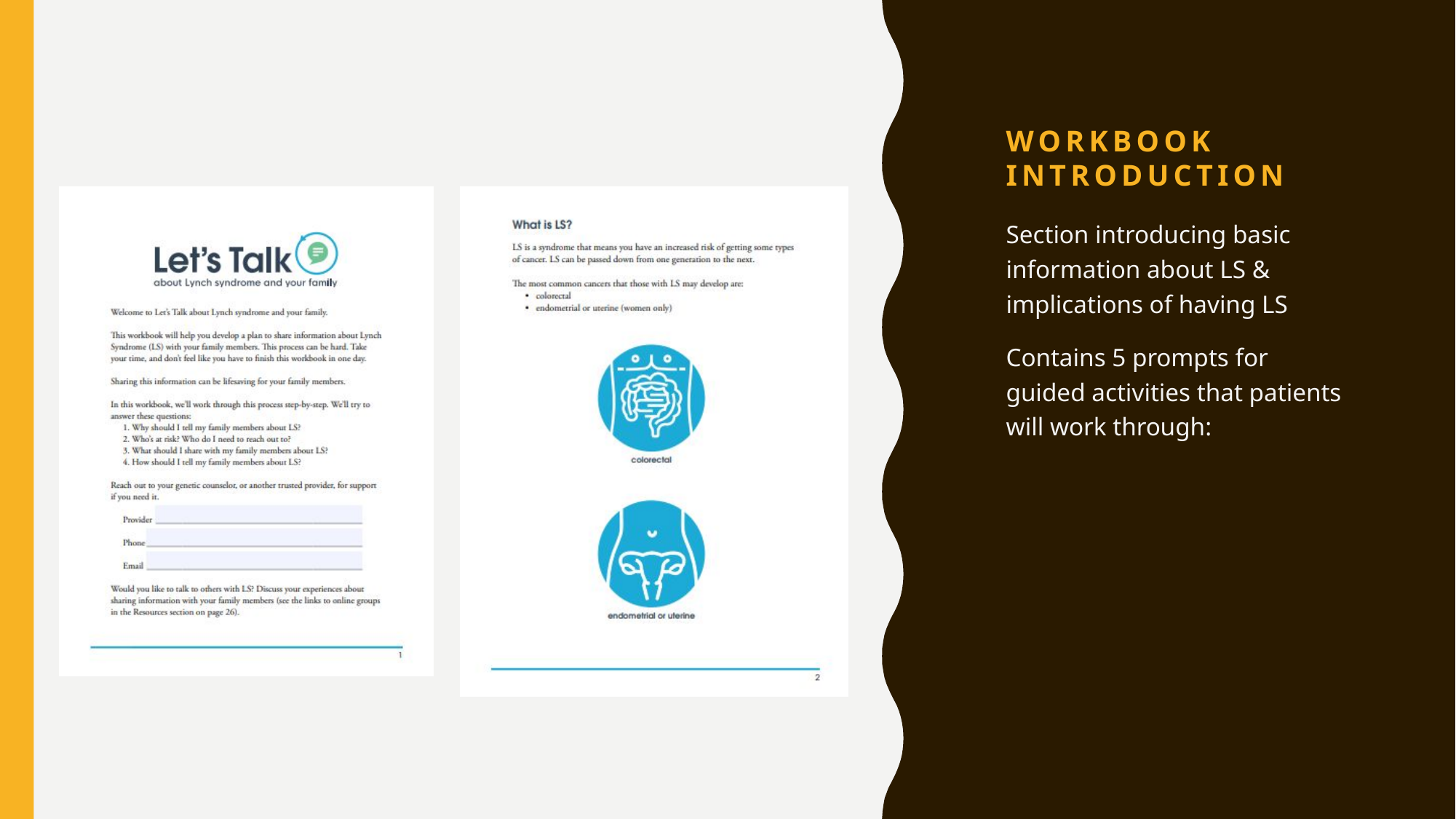

# Workbook introduction
Section introducing basic information about LS & implications of having LS
Contains 5 prompts for guided activities that patients will work through:

## Slide 6
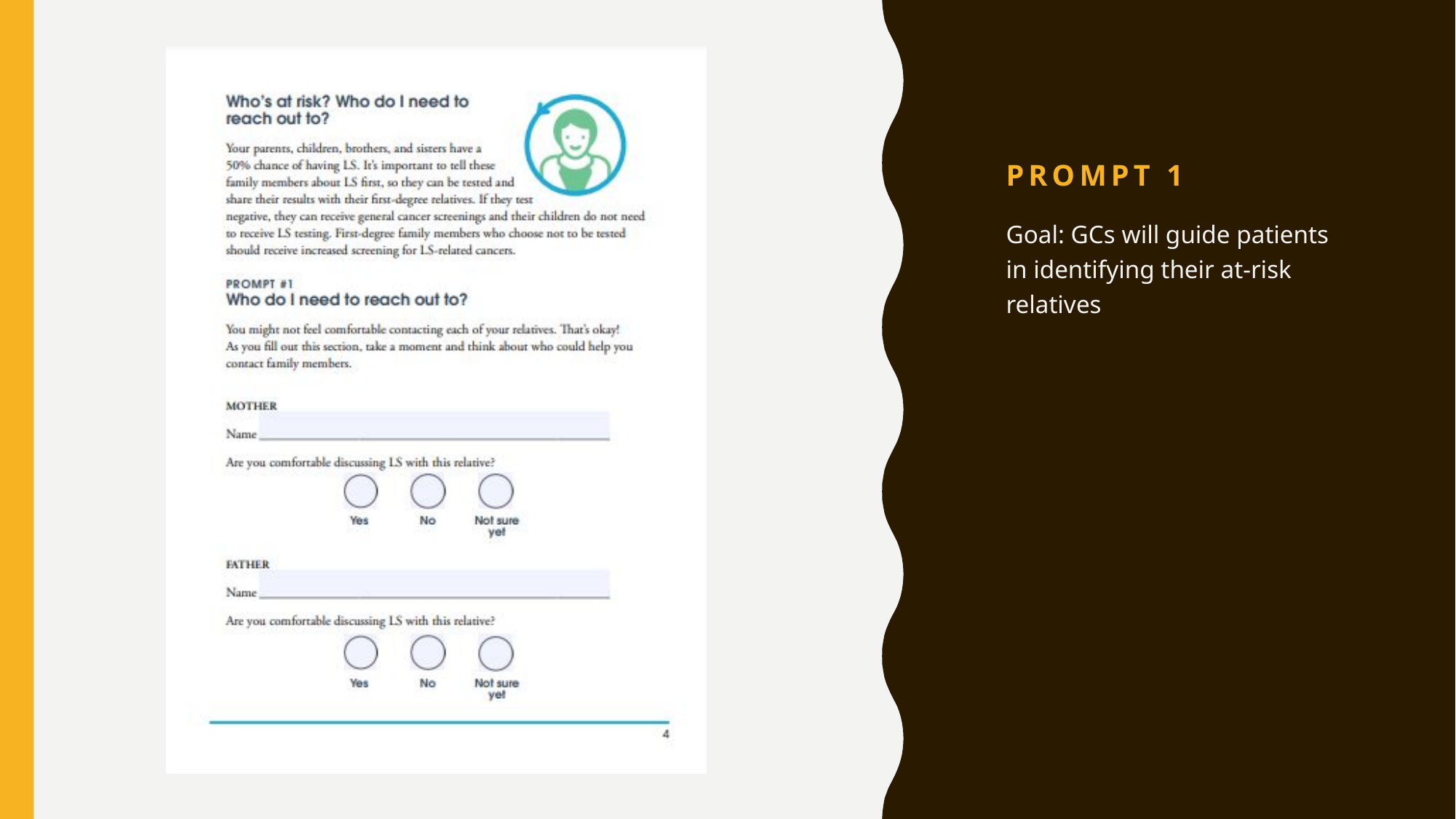

# Prompt 1
Goal: GCs will guide patients in identifying their at-risk relatives

## Slide 7
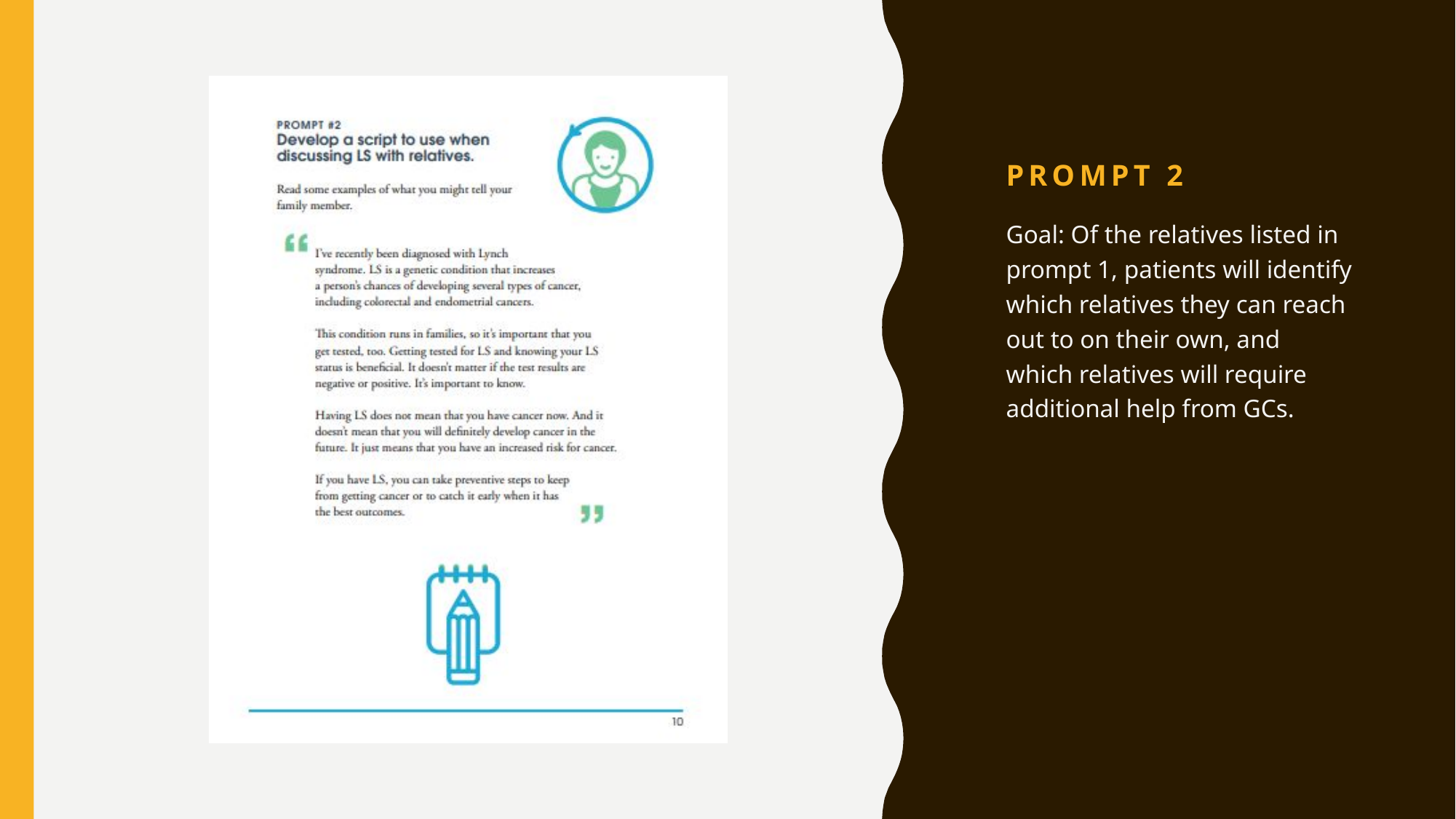

# Prompt 2
Goal: Of the relatives listed in prompt 1, patients will identify which relatives they can reach out to on their own, and which relatives will require additional help from GCs.

## Slide 8
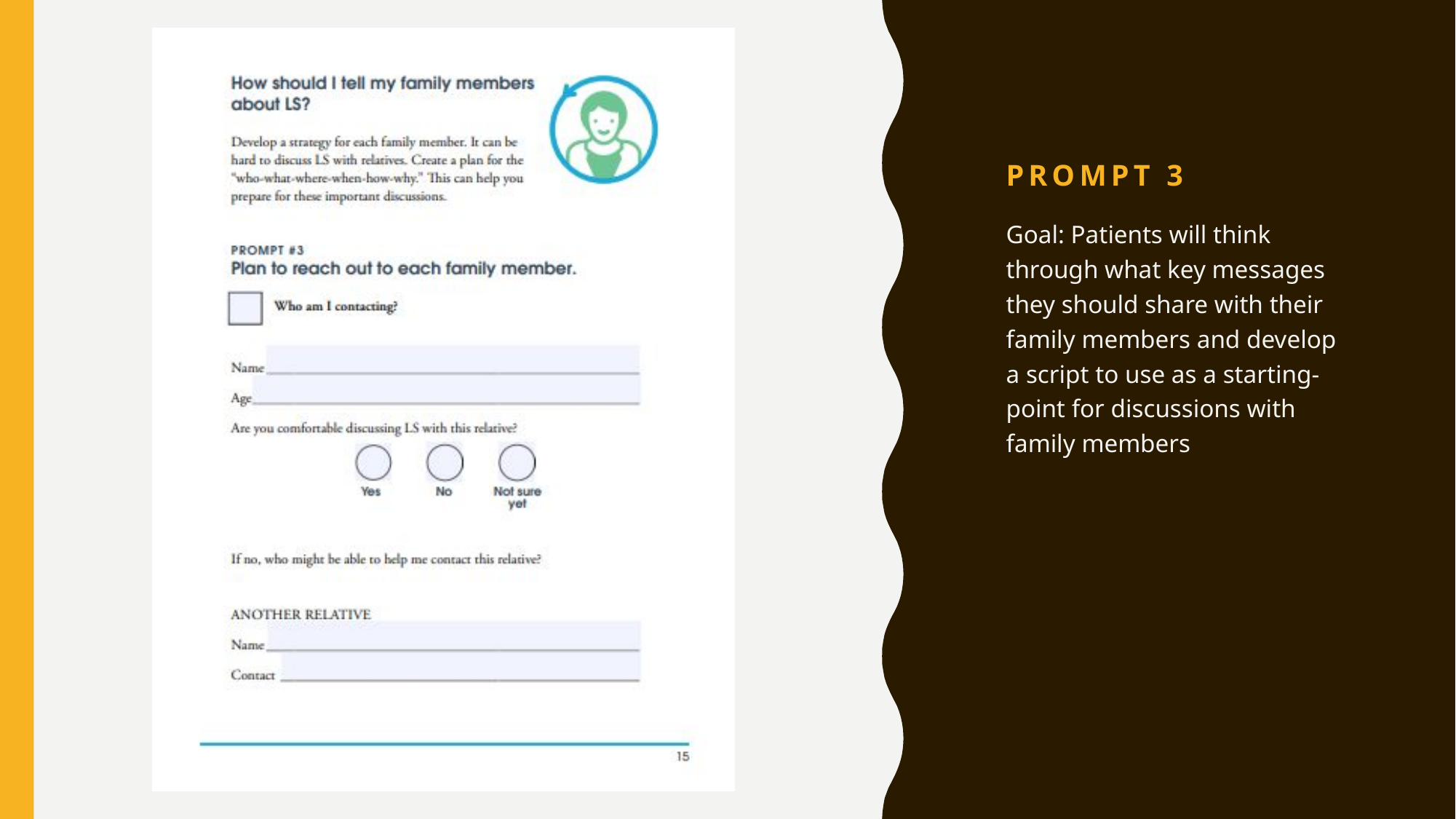

# Prompt 3
Goal: Patients will think through what key messages they should share with their family members and develop a script to use as a starting-point for discussions with family members

## Slide 9
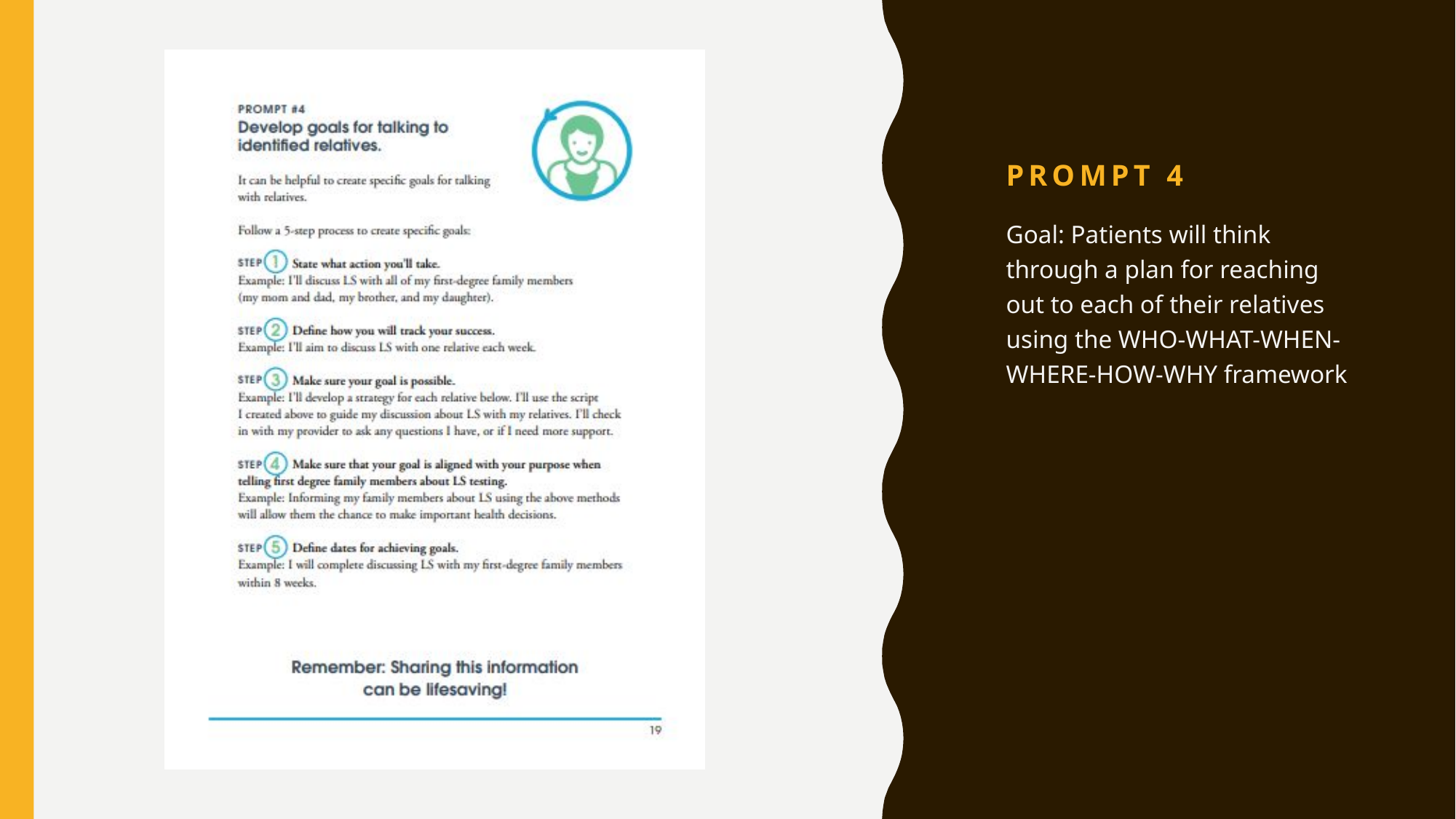

# Prompt 4
Goal: Patients will think through a plan for reaching out to each of their relatives using the WHO-WHAT-WHEN-WHERE-HOW-WHY framework

## Slide 10
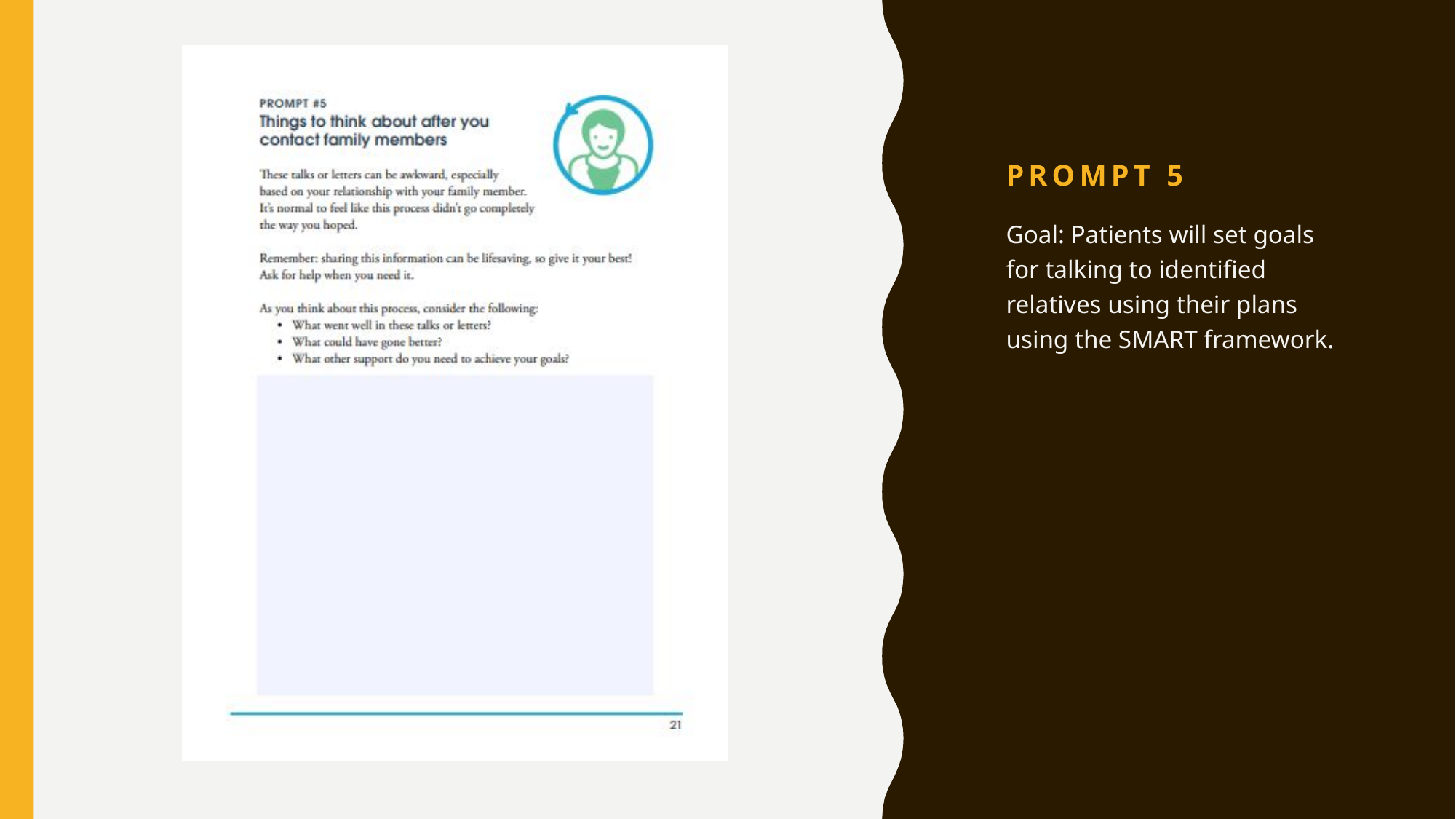

# Prompt 5
Goal: Patients will set goals for talking to identified relatives using their plans using the SMART framework.

## Slide 11
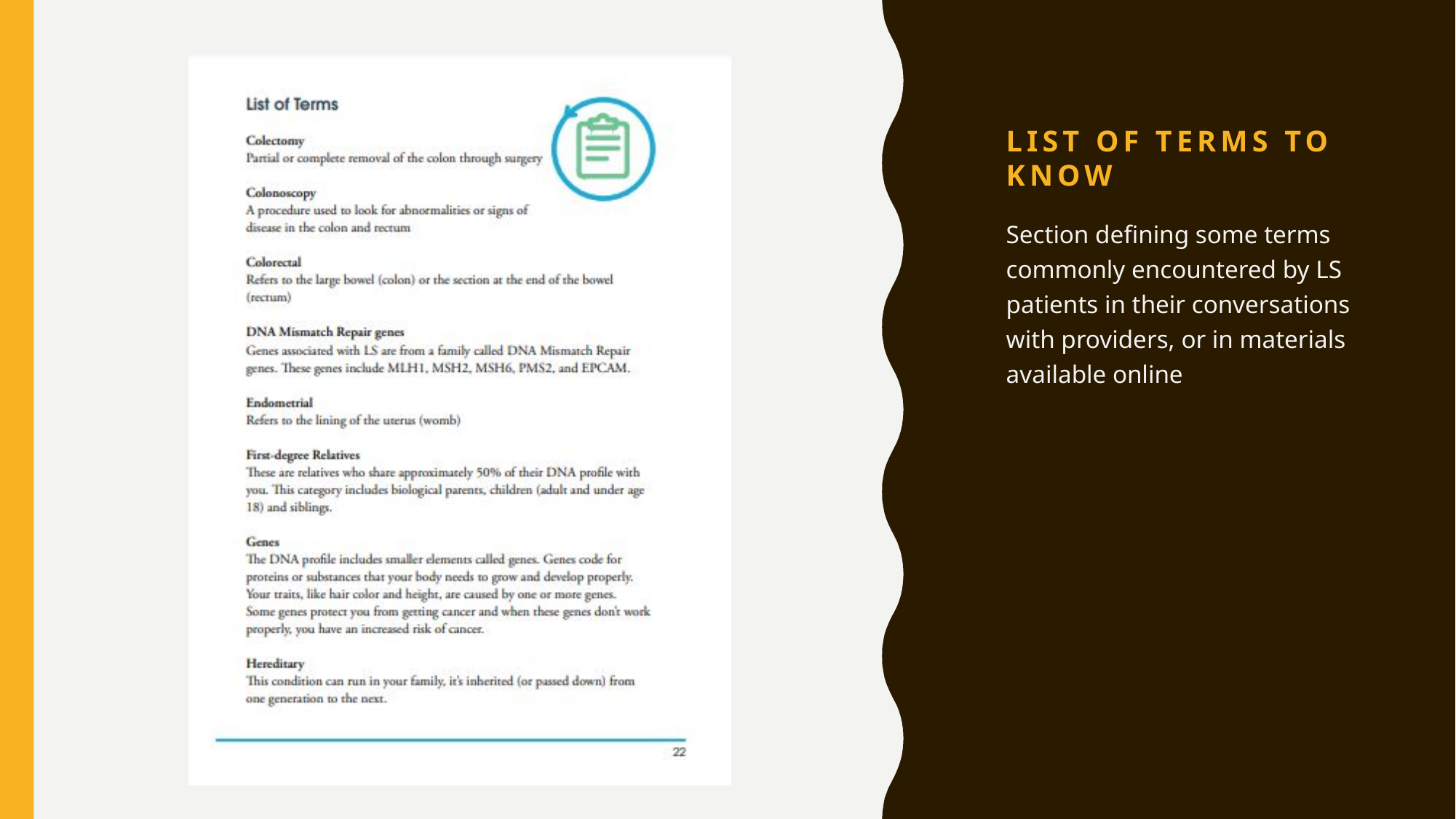

# LIST OF TERMS TO KNOW
Section defining some terms commonly encountered by LS patients in their conversations with providers, or in materials available online

## Slide 12
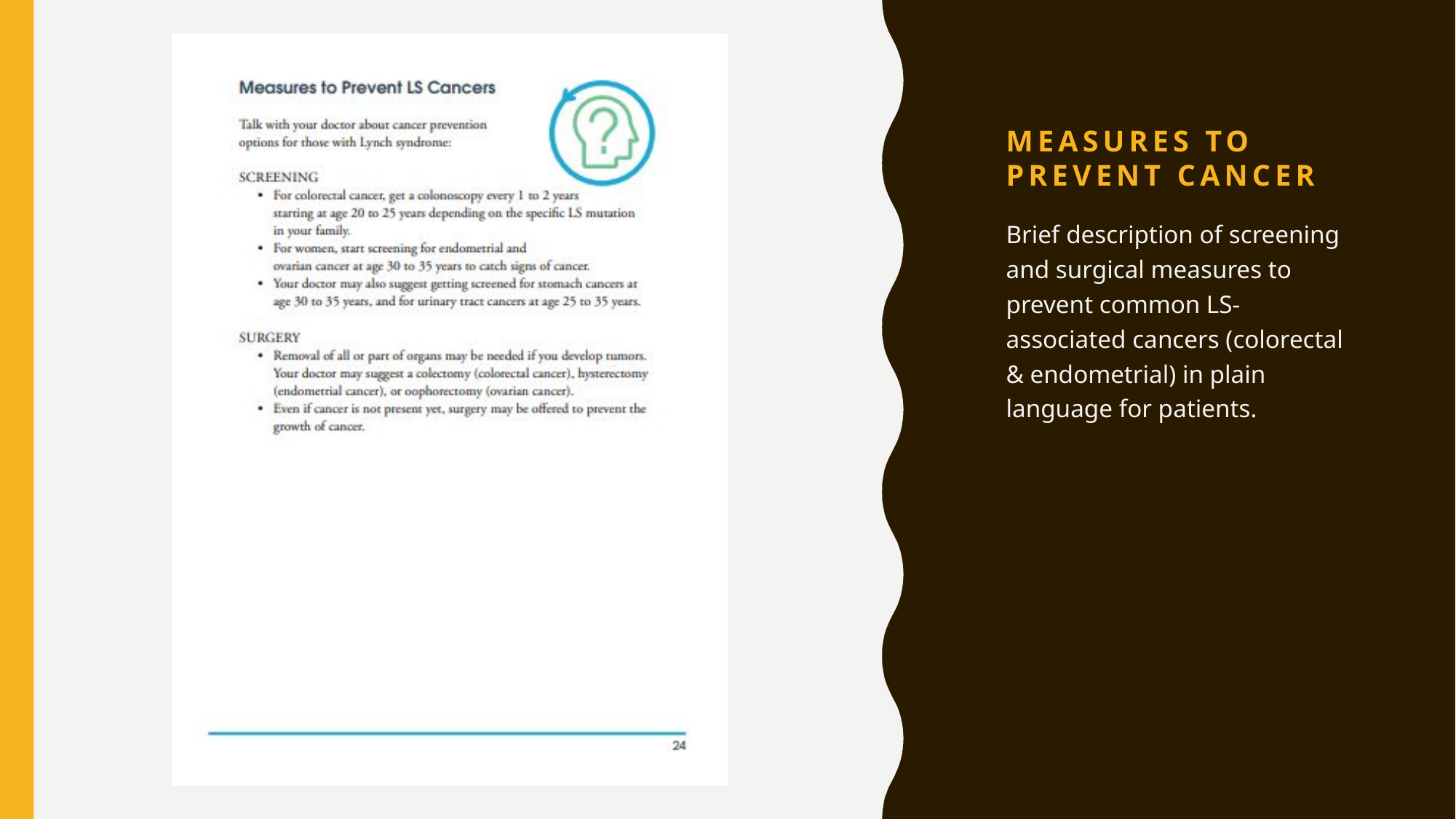

# MEASURES TO PREVENT CANCER
Brief description of screening and surgical measures to prevent common LS-associated cancers (colorectal & endometrial) in plain language for patients.

## Slide 13
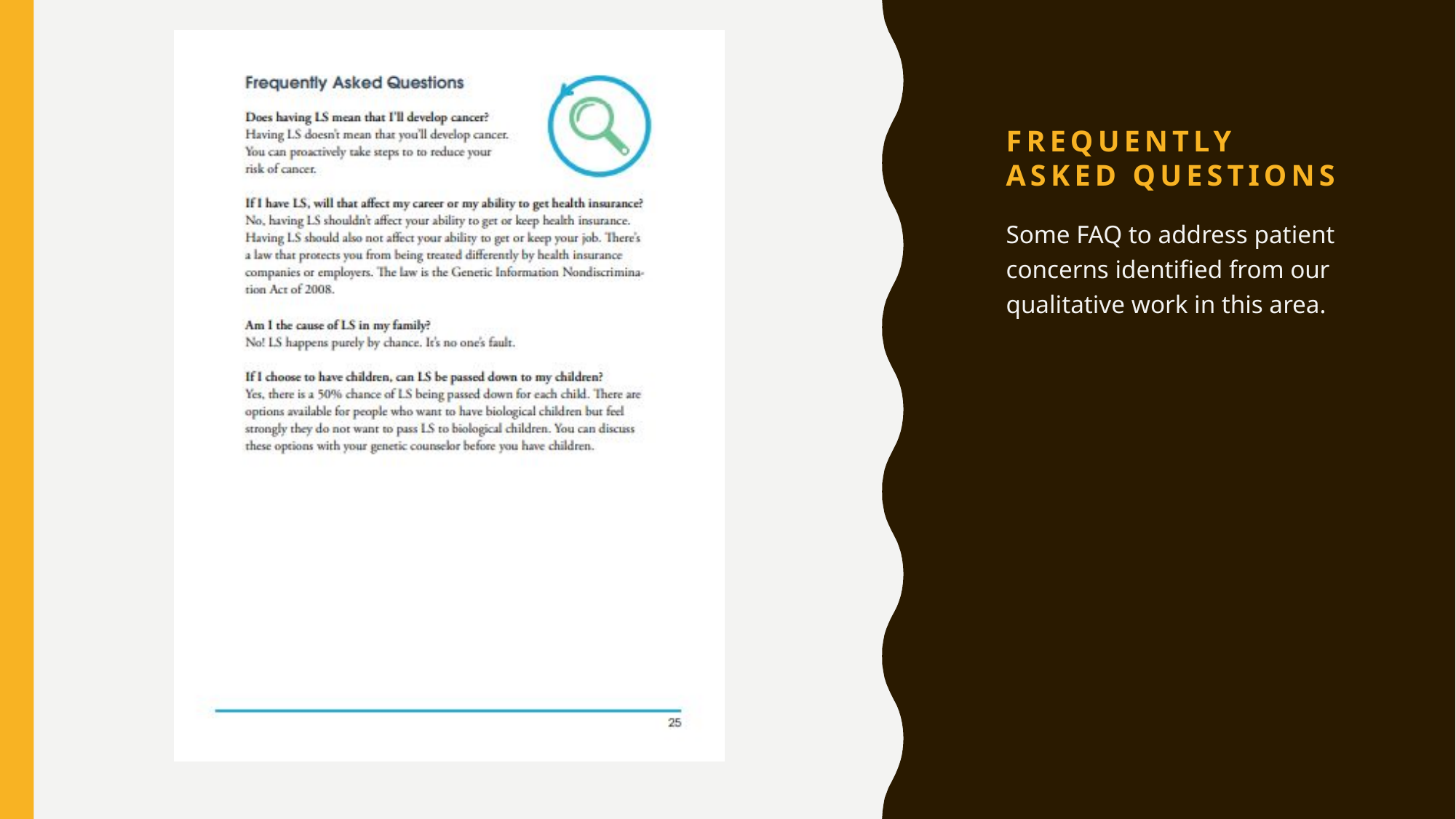

# FREQUENTLY ASKED QUESTIONS
Some FAQ to address patient concerns identified from our qualitative work in this area.

## Slide 14
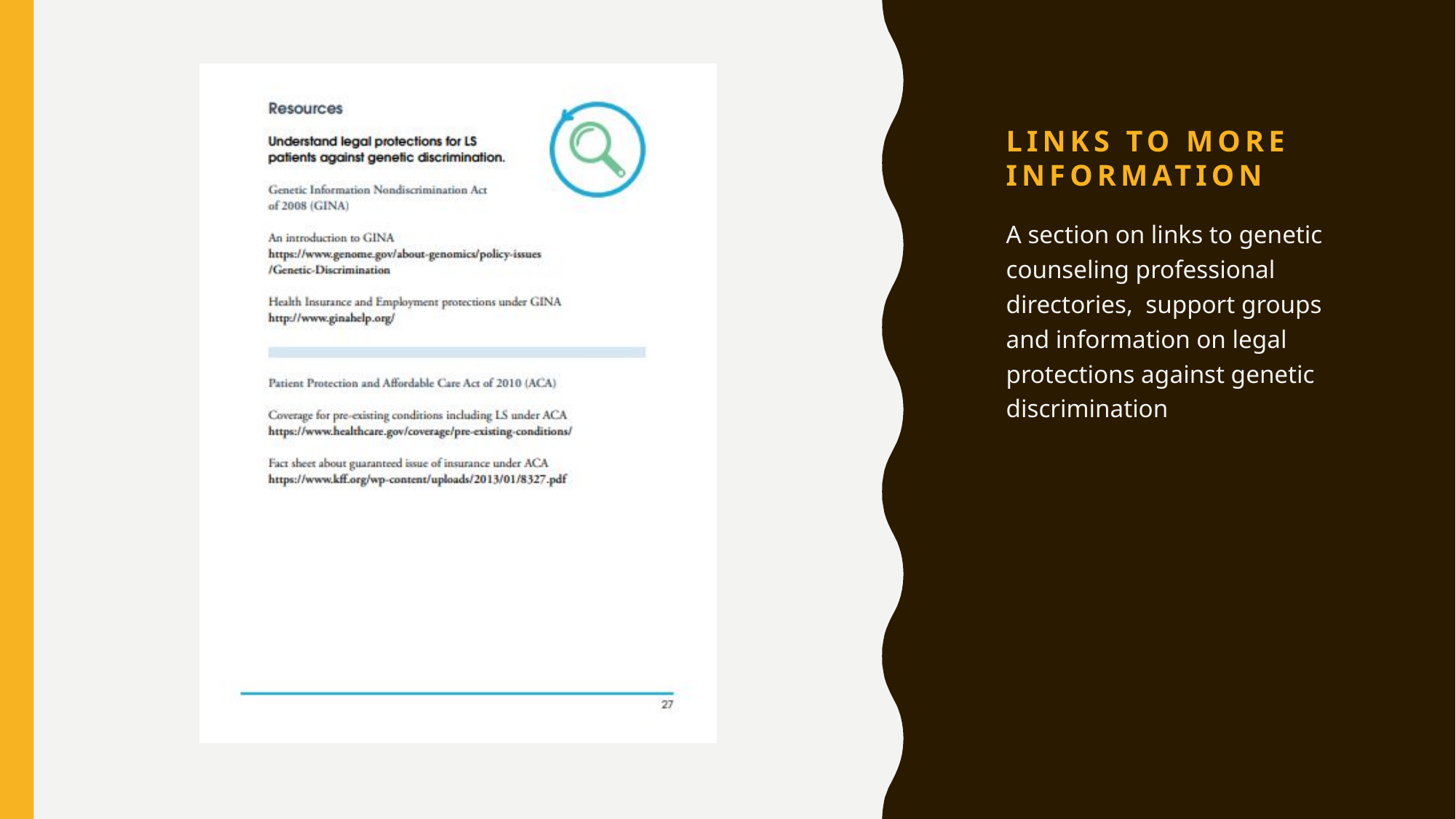

# LINKS TO MORE INFORMATION
A section on links to genetic counseling professional directories, support groups and information on legal protections against genetic discrimination

## Slide 15
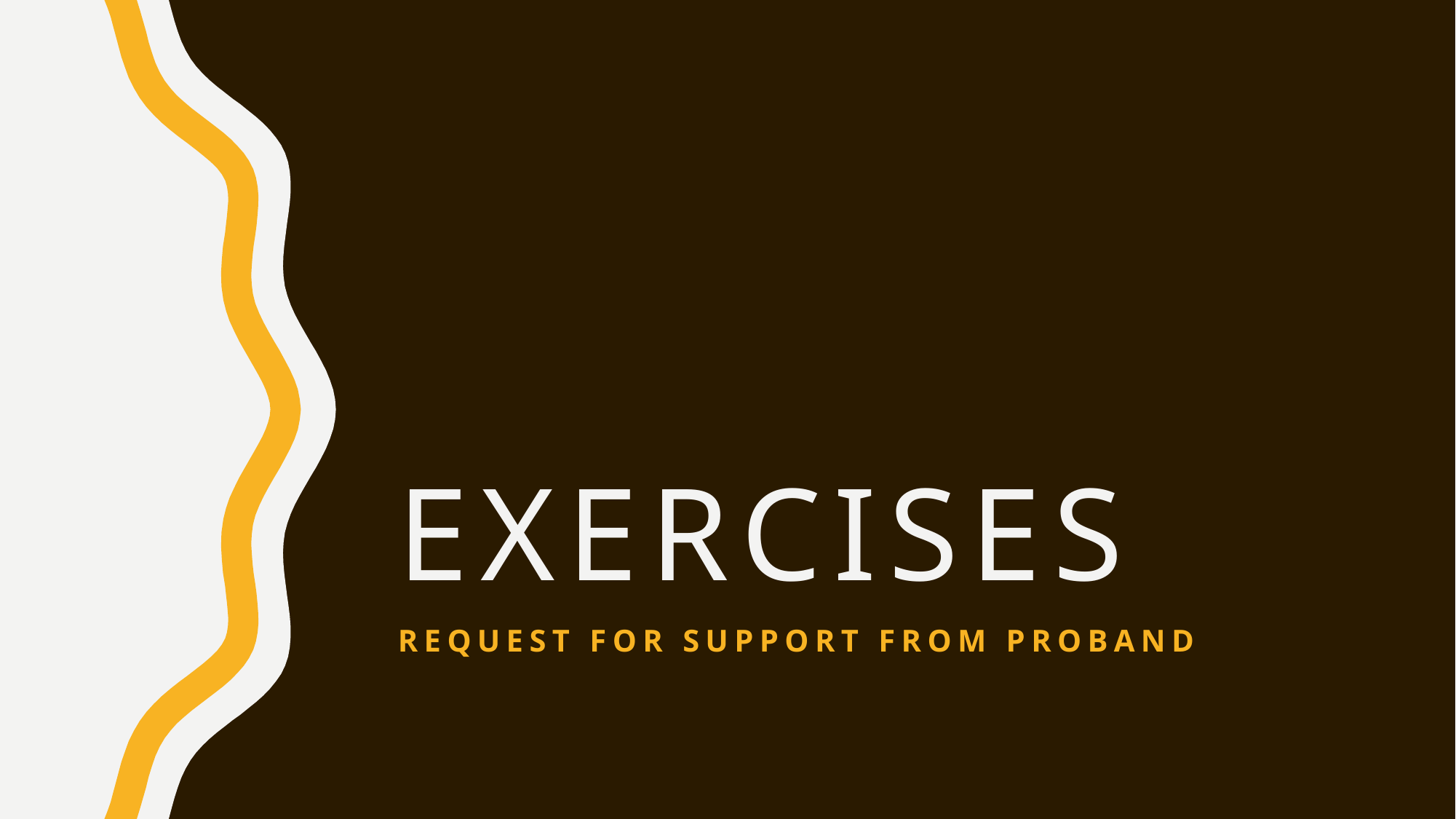

# EXercises
Request for support from proband

## Slide 16
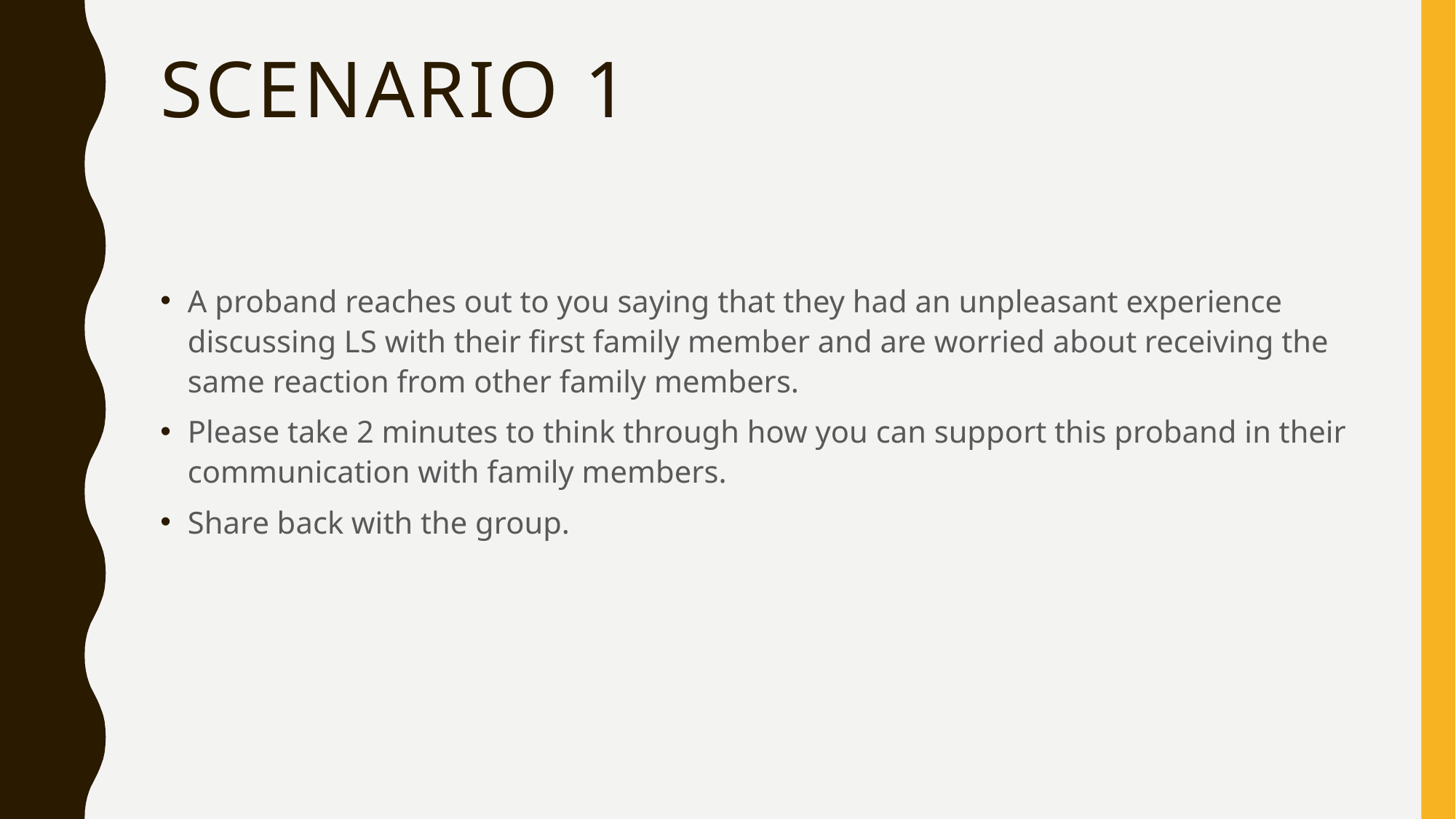

# Scenario 1
A proband reaches out to you saying that they had an unpleasant experience discussing LS with their first family member and are worried about receiving the same reaction from other family members.
Please take 2 minutes to think through how you can support this proband in their communication with family members.
Share back with the group.

## Slide 17
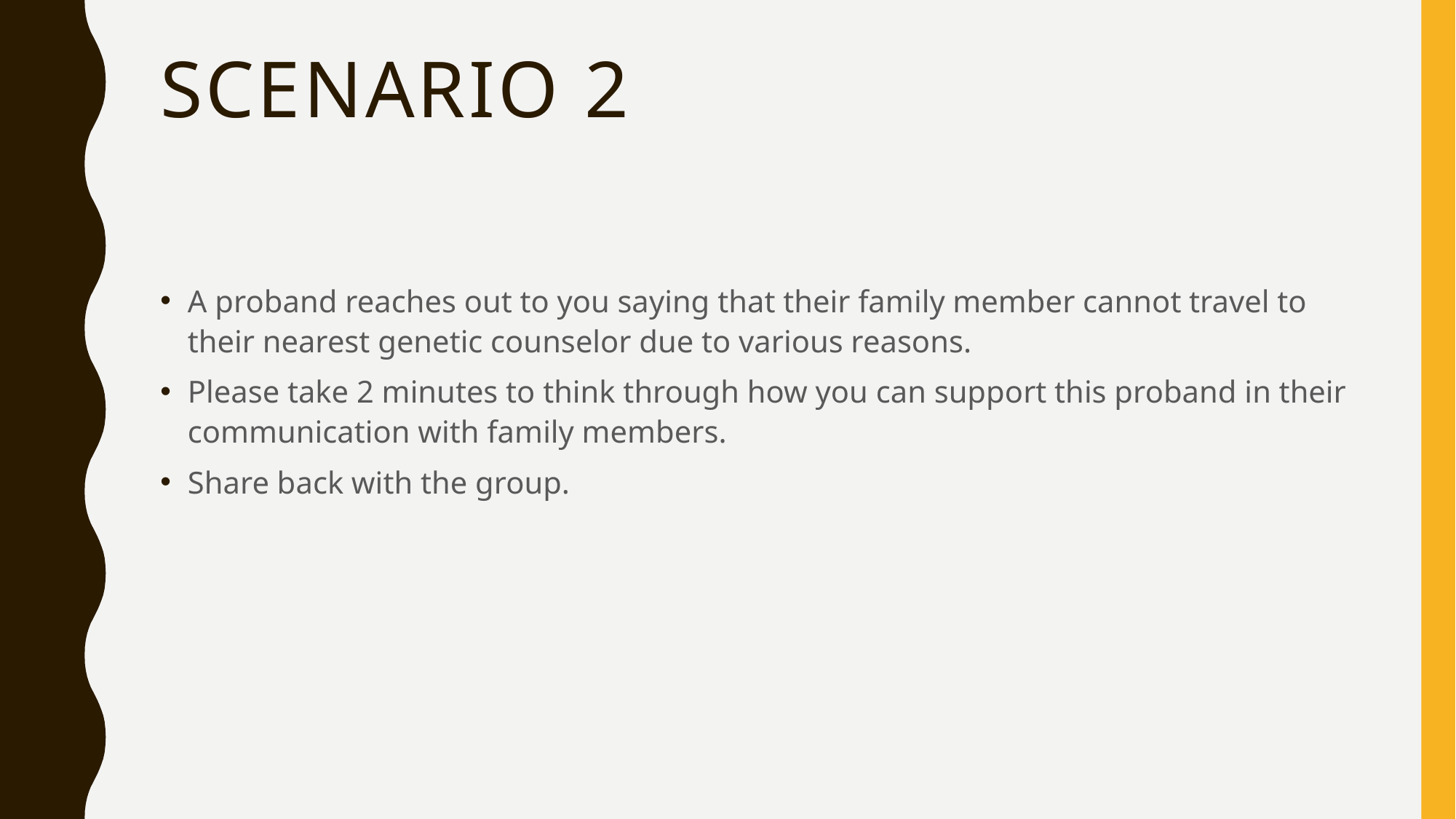

# Scenario 2
A proband reaches out to you saying that their family member cannot travel to their nearest genetic counselor due to various reasons.
Please take 2 minutes to think through how you can support this proband in their communication with family members.
Share back with the group.

## Slide 18
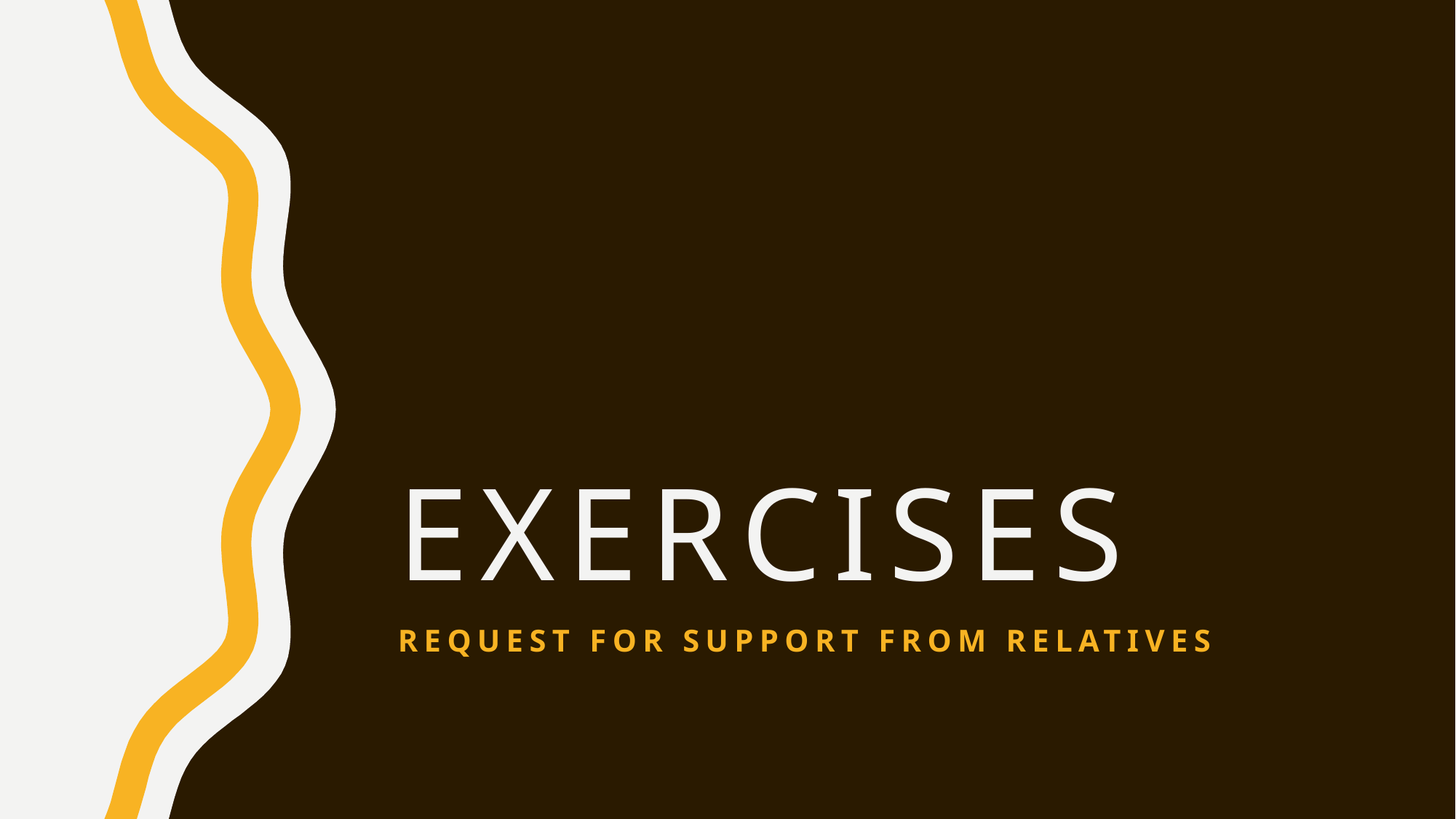

# Exercises
Request for support from relatives

## Slide 19
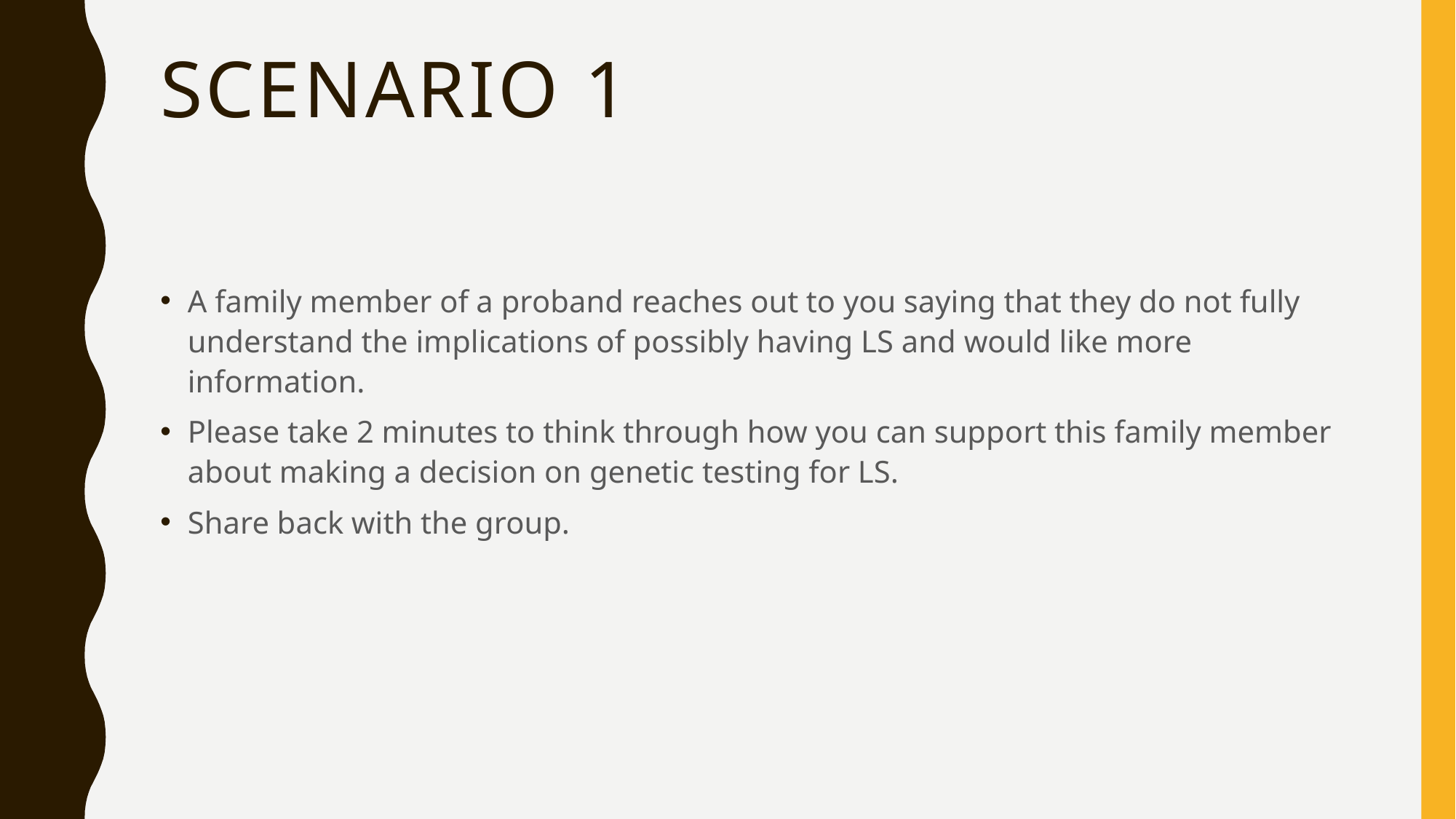

# scenario 1
A family member of a proband reaches out to you saying that they do not fully understand the implications of possibly having LS and would like more information.
Please take 2 minutes to think through how you can support this family member about making a decision on genetic testing for LS.
Share back with the group.

## Slide 20
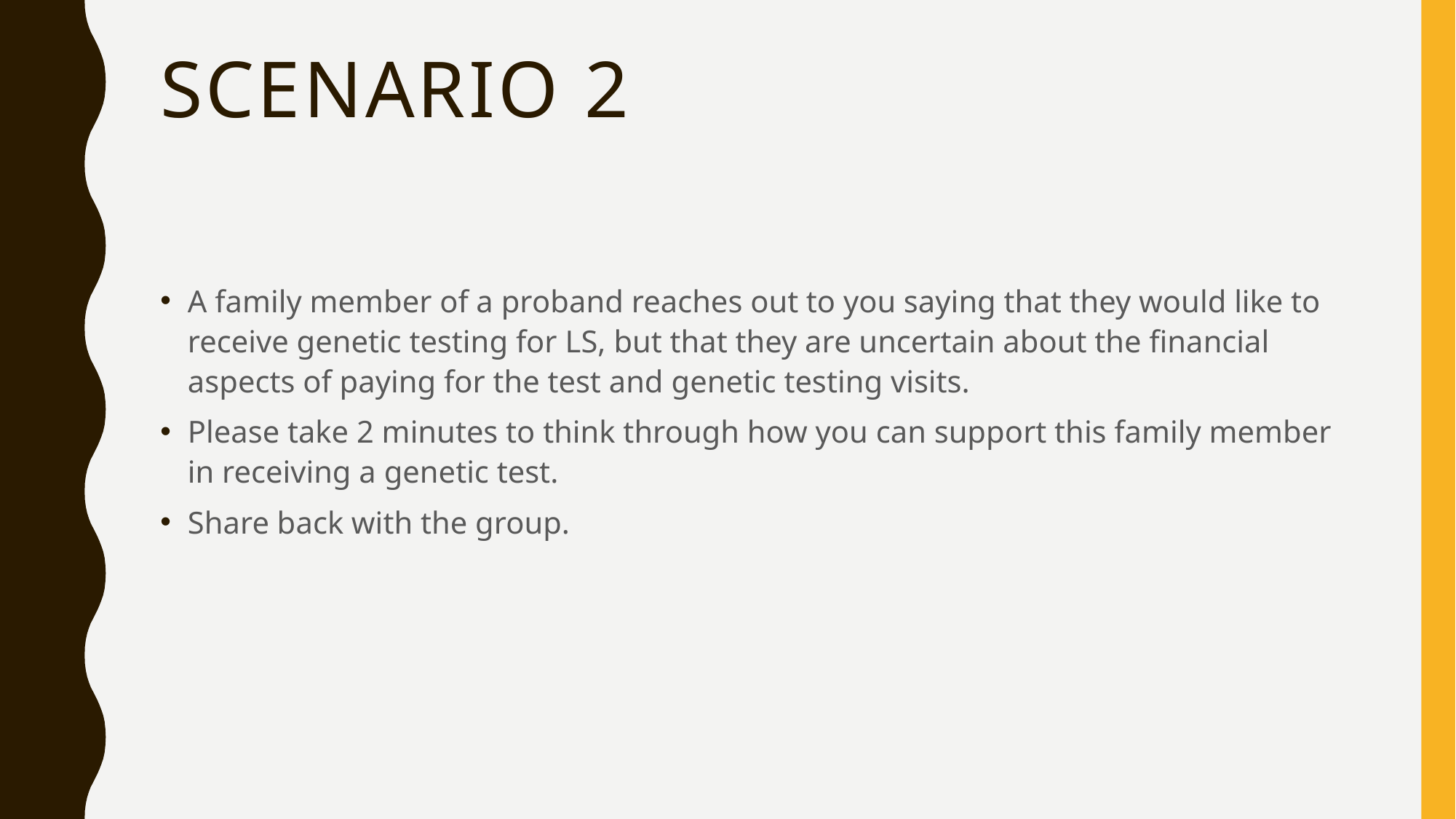

# scenario 2
A family member of a proband reaches out to you saying that they would like to receive genetic testing for LS, but that they are uncertain about the financial aspects of paying for the test and genetic testing visits.
Please take 2 minutes to think through how you can support this family member in receiving a genetic test.
Share back with the group.

## Slide 21
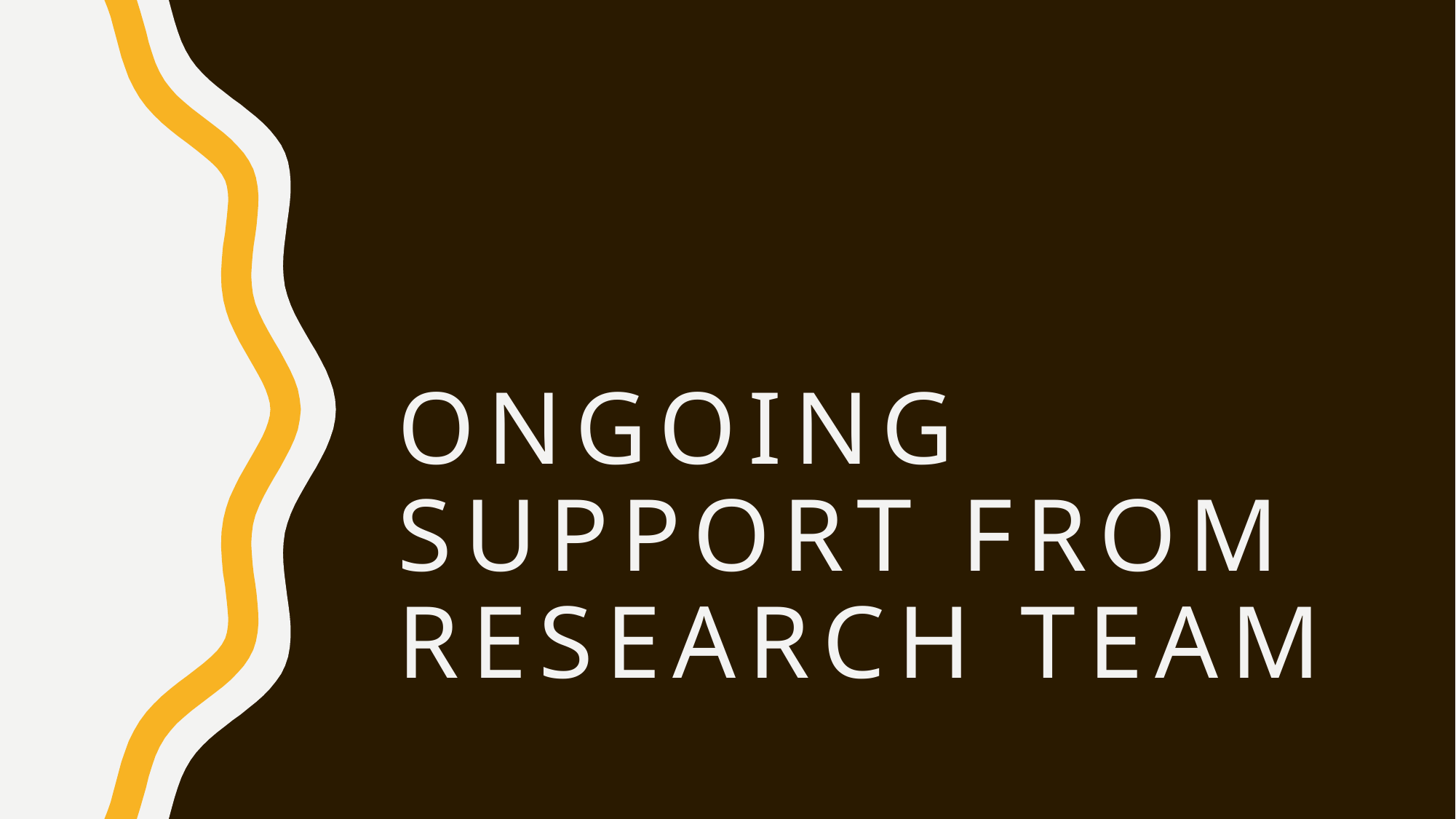

# Ongoing support from research team

## Slide 22
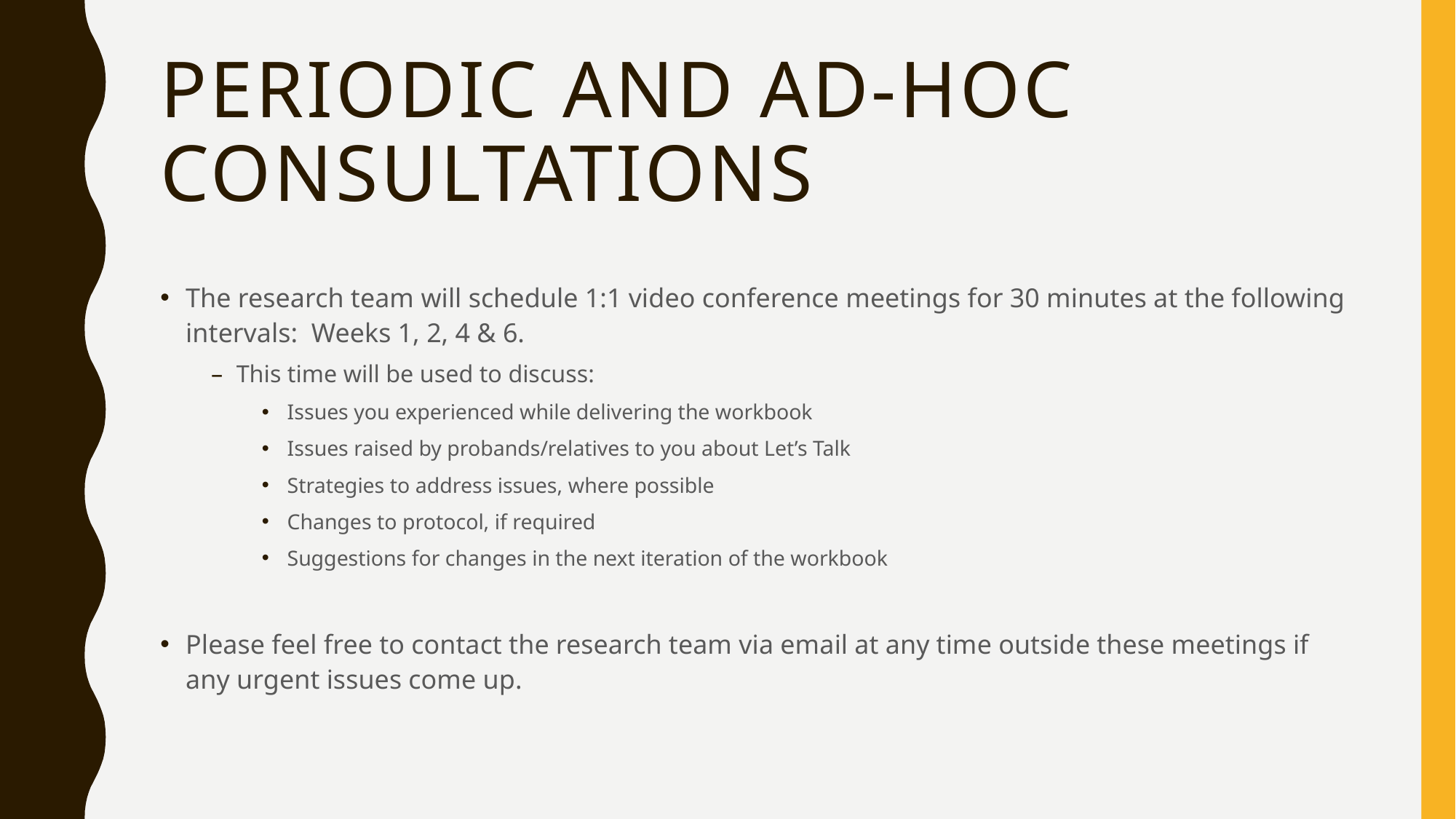

# PERIODIC AND AD-hoc consultations
The research team will schedule 1:1 video conference meetings for 30 minutes at the following intervals: Weeks 1, 2, 4 & 6.
This time will be used to discuss:
Issues you experienced while delivering the workbook
Issues raised by probands/relatives to you about Let’s Talk
Strategies to address issues, where possible
Changes to protocol, if required
Suggestions for changes in the next iteration of the workbook
Please feel free to contact the research team via email at any time outside these meetings if any urgent issues come up.

## Slide 23
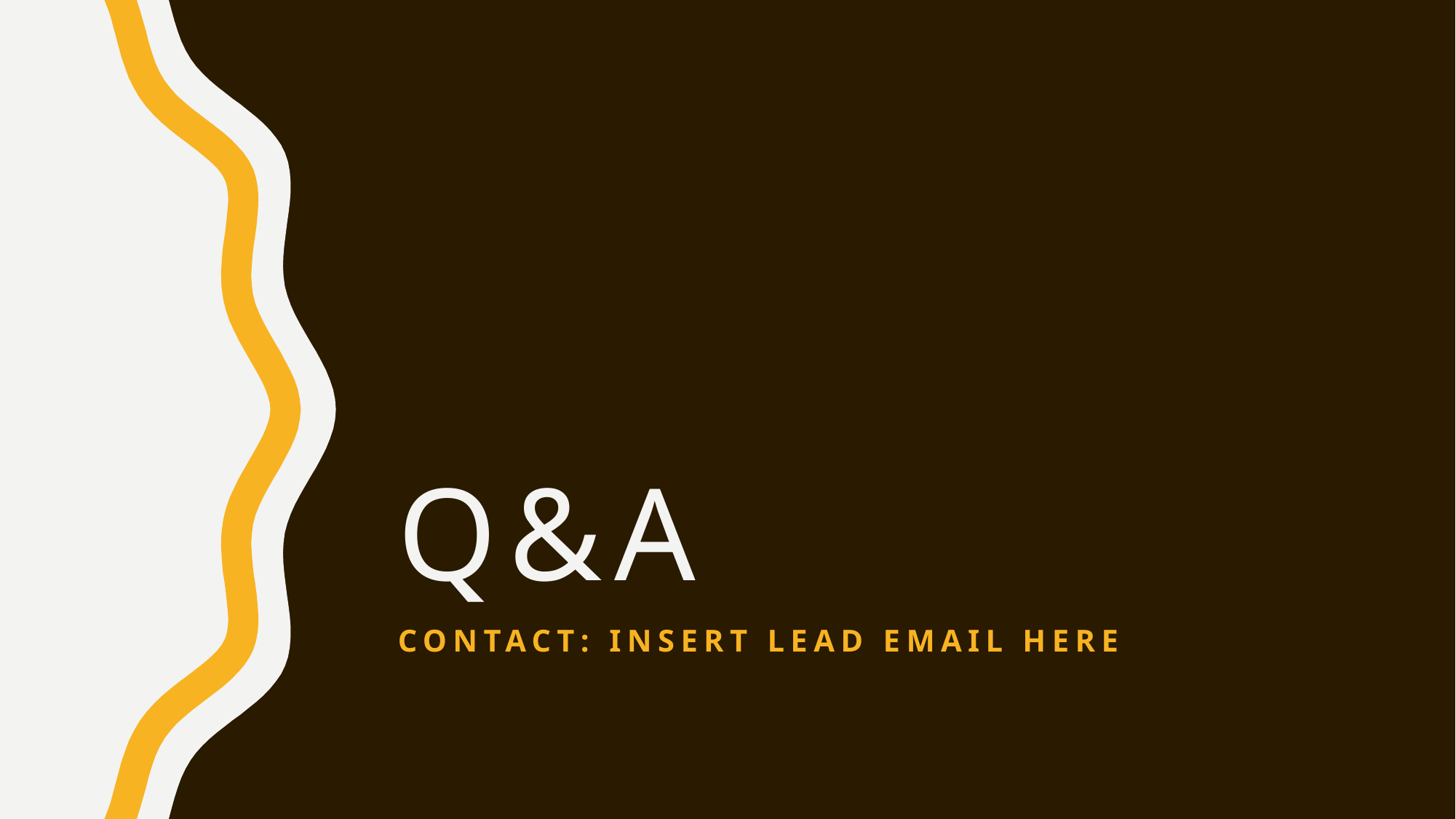

# Q&A
contact: INSERT Lead Email Here
